# Analysis of crystallographic phase retrieval using iterative projection algorithms

**DOI:** 10.1107/S2059798324009902

**Published:** 2024-10-23

**Authors:** Michael J. Barnett, Rick P. Millane, Richard L. Kingston

**Affiliations:** a School of Biological Sciences University of Auckland Auckland New Zealand; b Computational Imaging Group, Department of Electrical and Computer Engineering University of Canterbury Christchurch New Zealand; Lund University, Sweden

**Keywords:** *ab initio* phase determination, crystallographic imaging, iterative projection algorithms, nonconvex constraint-satisfaction problems

## Abstract

For protein crystals in which more than two thirds of the volume is occupied by solvent, the featureless nature of the solvent region often generates a constraint that is powerful enough to allow direct phasing of X-ray diffraction data. Practical implementation relies on the use of iterative projection algorithms with good global convergence properties to solve the difficult nonconvex phase-retrieval problem. In this paper, some aspects of phase retrieval using iterative projection algorithms are systematically explored, where the diffraction data and density-value distributions in the protein and solvent regions provide the sole constraints. The analysis is based on the addition of random error to the phases of previously determined protein crystal structures, followed by evaluation of the ability to recover the correct phase set as the distance from the solution increases. The properties of the difference-map (DM), relaxed–reflect–reflect (RRR) and relaxed averaged alternating reflectors (RAAR) algorithms are compared. All of these algorithms prove to be effective for crystallographic phase retrieval, and the useful ranges of the adjustable parameter which controls their behavior are established. When these algorithms converge to the solution, the algorithm trajectory becomes stationary; however, the density function continues to fluctuate significantly around its mean position. It is shown that averaging over the algorithm trajectory in the stationary region, following convergence, improves the density estimate, with this procedure outperforming previous approaches for phase or density refinement.

## Abstract 2

The behavior of iterative projection algorithms for crystallographic phase retrieval is systematically investigated. It is shown that information present in the algorithm trajectory following convergence can be exploited to improve the final density estimate.

## 1. Introduction

Direct phase determination using the diffraction amplitude data alone has been a long-sought goal in protein crystallo­graphy. This problem admits no solution unless something is known about the density function within the crystal (Millane & Arnal, 2015 ▸; Millane, 1990 ▸, 2023 ▸). Recently, it has been demonstrated that the largely featureless nature of the solvent region generates a constraint that is powerful enough to directly determine phases for protein crystals with a high solvent content (Liu *et al.*, 2012 ▸; He & Su, 2015 ▸; Kingston & Millane, 2022 ▸). Unlike traditional direct methods, which rest on the atomicity of the image (Hauptman, 1998 ▸), these new direct phasing techniques work at more modest resolution. Key to these methods is the use of iterative projection algorithms with good global convergence properties (Marchesini, 2007 ▸; Millane & Lo, 2013 ▸; Millane, 2023 ▸) to perform phase retrieval.

Fundamental to this approach is the treatment of crystallographic phase retrieval as a constraint-satisfaction problem. In this formulation, the problem is to find a density function that satisfies a number of constraints in both real and reciprocal space. In reciprocal space, the constraint is that the structure-factor amplitudes must equal their experimentally measured values. In real space, various constraints are possible. In addition to the enforcement of a constant density in the solvent region (a solvent-flatness constraint; Bricogne, 1974 ▸; Hendrickson, 1981 ▸; Wang, 1985 ▸), these might include the enforcement of a suitable prior for the density-value distribution in the protein region (a histogram-equivalence constraint; Harrison, 1988 ▸; Lunin, 1988 ▸; Zhang & Main, 1990 ▸), or the enforcement of density equivalence when multiple copies of a molecule are present in the asymmetric unit of the crystal (a symmetry constraint; Lawrence, 1991 ▸; Rossmann, 1995 ▸; Vellieux & Read, 1997 ▸; Kleywegt & Read, 1997 ▸). In fact, in principle any *a priori* property of the density can be incorporated as a constraint. If the constraints, taken together, are sufficiently restrictive, then only one density will simultaneously satisfy all of them, and the solution to the phase-retrieval problem is unique (Millane & Arnal, 2015 ▸). If the available constraints are not sufficiently powerful, then they will be satisfied by multiple density functions and the solution is not unique. In this case, direct phase retrieval will not be possible.

Assuming that sufficiently powerful constraints are available, the problem of finding a density satisfying all of the constraints remains. Iterative projection algorithms (IPAs), which are an evolution of traditional electron-density modification techniques (Podjarny, 1987 ▸; Cowtan & Zhang, 1999 ▸), provide an effective way to approach this problem. In these algorithms, the density is iteratively adjusted based on the constraints existing in real and reciprocal space, with the objective of converging to the solution. However, a primary difficulty with this approach is that the reciprocal-space constraint, involving the structure-factor amplitudes, is non­convex (for a definition and discussion, see Millane & Lo, 2013 ▸). This nonconvexity makes the associated optimization problem very difficult. An iterative projection algorithm in which the constraints are alternately and exactly satisfied on every iteration (equivalent to density modification, as it was originally conceived; Bricogne, 1974 ▸) will only converge to the solution when initiated with a density (or equivalently with a phase set) that is close to the solution. Therefore, traditional density modification, while very powerful for improving experimentally determined phases that are substantially correct, is not useful for direct phasing where the starting densities (or phase sets) are fully randomized.

Fortunately, there are other IPAs that are more effective in finding the solution to difficult nonconvex constraint-satisfaction problems (Elser, 2003 ▸; Millane & Lo, 2013 ▸; Millane, 2023 ▸). These algorithms have good global (as opposed to local) convergence properties and thus are potentially effective for phase retrieval without any prior phase information (*i.e.* starting from a random density). We and others have demonstrated the potential of these algorithms for direct phasing in protein crystallography (He & Su, 2015 ▸; Kingston & Millane, 2022 ▸). In particular, we developed a practical method to directly phase diffraction data from high-solvent-content protein crystals (Kingston & Millane, 2022 ▸) using the difference-map (DM) IPA (Elser, 2003 ▸). A two-stage procedure was found to be most computationally efficient, in which an approximate molecular envelope is first determined at low resolution, with knowledge of the envelope subsequently exploited to aid phase retrieval using all data. The DM algorithm is used in both stages. The performance of the procedure was optimized empirically through application to previously determined protein crystal structures.

The DM algorithm is, however, only one of a number of IPAs that have been used to solve difficult noncomplex optimization problems, with some other specific algorithms being the relaxed–reflect–reflect (RRR) algorithm (Elser *et al.*, 2018 ▸) and the relaxed averaged alternating reflections (RAAR) algorithm (Luke, 2005 ▸). The performance of these latter algorithms for crystallographic phase retrieval is untested and cannot be predicted from existing theory; however, differences in detailed behavior from the DM algorithm are expected.

In this paper, we conduct a comprehensive survey of the properties and behavior of IPAs for phase retrieval in protein crystallography, using solvent flatness and histogram equivalence as the real-space constraints. In the first part of the study, we compare the behavior and performance of the DM, RRR and RAAR algorithms as a function of their adjustable algorithm parameter. We do this by simulation, introducing random error into the phases of previously determined protein crystal structures and testing the ability of the algorithms to return to the solution as the magnitude of the error increases. Subsequently, we perform an analysis of algorithm behavior in the Fourier domain, examining the trajectories of individual Fourier coefficients as the algorithms progress. This analysis suggests a new and effective way to deploy these algorithms in which the information present in the algorithm trajectories following convergence is exploited to improve the final phase (or density) estimates.

The paper is structured as follows. In Section 2 we briefly review the algorithms used and the constraints employed. In Section 3 we describe the simulation strategy, error model, agreement measures and methods of analysis. In Sections 4, 5 and 6 we describe our results, while in Section 7 we summarize the findings and discuss the implications for the use of these algorithms for protein crystallographic phasing.

## 2. Algorithms and constraints

### 2.1. Iterative projection algorithms

As noted in Section 1, IPAs are iterative schemes, where at each iteration a density estimate is adjusted based on the various constraints, with the objective of moving the estimate to one that satisfies all of the constraints (*i.e.* one that lies in the intersection of the constraint sets). Such an estimate represents a valid solution to the constraint-satisfaction problem. For the purposes of describing these algorithms, the density function is represented as an *N*-dimensional vector, with each element of the vector associated with a point of the discrete 3D grid that samples the density in the asymmetric unit (or unit cell). The values carried in the vector may represent a surrogate function that does not actually correspond to the values of the density function. However, a density estimate can be calculated from the function carried in the vector. For this reason, the vector is referred to as the iterate. We denote the iterate at the *n*th iteration of the algorithm by **x**_*n*_.

One iteration of an IPA produces a new iterate **x**_*n*+1_, which is calculated from **x**_*n*_ using an update rule. The IPAs described here consider only two constraint sets. This is the norm, and is not generally restrictive in practice, as constraints can often be sensibly combined. As is usual, we consider one constraint in real space, denoted *A*, and one in reciprocal space, denoted *B*. The update rule can then be written as
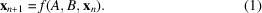


The function *f*(·) then defines the IPA. The update rule involves steps in which the iterate is adjusted so the corresponding density estimate is moved towards a position satisfying both sets of constraints. These steps involve ‘projections’ onto the constraints, which are adjustments that satisfy the constraints while minimizing change in the squared difference sense. The projection of the iterate **x** onto the constraint set *A* is formally written as *P*_*A*_**x**, with *P*_*A*_ representing the projection operation.

The projections usually correspond to the operations performed in conventional density modification. For example, projection onto a solvent-flatness constraint corresponds to setting the iterate (or some function derived from the iterate) to a constant value at all points within the solvent region, while leaving the remaining points unchanged. The difference between different IPAs (and the difference from conventional density modification) lies in the way that the projections are subsequently incorporated into the update rule.

In this paper, we assess the performance of several different IPAs for phase retrieval. These are defined briefly below using a common notation, followed by a discussion of the constraints employed, which are common to all of the algorithms employed here. The reader is referred to Millane & Lo (2013 ▸), Marchesini (2007 ▸) and Millane (2023 ▸) for a general review of IPAs. Further details of our practical implementation of IPAs in a crystallographic setting are given in Kingston & Millane (2022 ▸).

It is worth pointing out that we do not consider one of the first, and arguably one of the most popular, phase-retrieval algorithms, the hybrid input–output (HIO) algorithm (Fienup, 1982 ▸). This is because the HIO algorithm accommodates only support and positivity constraints, cannot be couched in general as an IPA and does not have the general applicability of the algorithms that we consider here. While the HIO algorithm has been used successfully for crystallographic phase retrieval (Liu *et al.*, 2012 ▸; He & Su, 2015 ▸, 2018 ▸), we omit it from this study because of its fundamentally different character.

#### 2.1.1. The error-reduction (ER) or Gerchberg–Saxton algorithm

The error-reduction (ER) algorithm (Fienup, 1982 ▸; Gerchberg & Saxton, 1972 ▸) consists of sequentially applying the two projections *P*_*A*_ and *P*_*B*_ to complete one iteration of the algorithm (Fienup, 1982 ▸; Gerchberg & Saxton, 1972 ▸). The update rule is given by
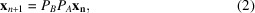
where *P*_*A*_ represents the projection onto the real-space constraints and *P*_*B*_ represents the projection onto the Fourier-space constraints.

It is immediately obvious that this algorithm corresponds to classical crystallographic density modification (Bricogne, 1974 ▸). The problem with this algorithm is that unless it is initiated close to the solution, it quickly converges to a density that does not satisfy both constraints, and so is not effective for *ab initio* phase retrieval. However, we include it as a control in some of our computational experiments.

#### 2.1.2. The difference-map (DM) algorithm

The difference-map (DM) algorithm (Elser, 2003 ▸) is designed to avoid stagnation at a nonsolution and continues to explore the parameter space if the constraints are not both satisfied. The update rule is given by
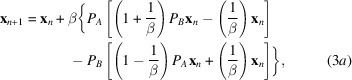
where β ∈ (−1, 1) is an adjustable parameter. Changing the sign of β effectively changes the role of the two constraints in the update rule.

For the DM algorithm, **x**_*n*_ is a surrogate function which is not itself an estimate of the density. However, each time the update rule is evaluated, two solution estimates are generated, which fully satisfy the constraints *A* or *B*, respectively. These are given by
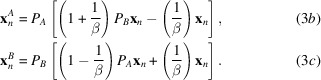


Prior to convergence these two solution estimates are not generally equal. However, when the iterate becomes stationary (*i.e.***x**_*n*+1_ ≅ **x**_*n*_) inspection of equation (3*a*) shows that the two estimates in equations (3*b*) and (3*c*) must become equivalent and they represent a potential solution to the problem, since they satisfy both sets of constraints.

In evaluating the performance of the DM algorithm we monitored agreement between the known solution and the solution estimate (equation 3*c*[Disp-formula fd4]), which exactly satisfies the Fourier-space constraints.

#### 2.1.3. The relaxed–reflect–reflect (RRR) algorithm

The relaxed–reflect–reflect (RRR) algorithm (Elser *et al.*, 2018 ▸) is defined by the update rule
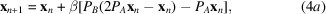
where β ∈ (0, 2) is an adjustable parameter. As with the DM algorithm, two solution estimates can be calculated at each iteration which fully satisfy the constraints *A* or *B*, respectively. These are given by
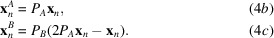


As with the DM algorithm, the update rule involves the difference of these estimates, and the estimates become equivalent when the iterate becomes stationary (**x**_*n*+1_ ≅ **x**_*n*_). A significant advantage of the RRR algorithm over the DM algorithm is that the computational cost per iteration is halved. This can be seen by comparing equations (3*a*) and (4*a*). It is worth noting that the RRR algorithm with β = 1 is identical to the Douglas–Rachford algorithm (Douglas & Rachford, 1956 ▸).

Inspection of equation (4*a*) shows that interchanging the projections *P*_*A*_ and *P*_*B*_ in the update rule results in a different algorithm, which cannot be obtained by manipulating β, as is the case for the DM algorithm. Making this change still results in an RRR algorithm, but the behavior of the algorithm is expected to be different. To distinguish the two cases, we refer to this second case as the reversed RRR (revRRR) algorithm, with update rule
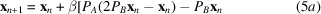
and the two solution estimates given by
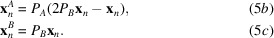


In evaluating the performance of the RRR or revRRR algorithm we monitored the agreement between the known solution and the solution estimates given by equations (4*c*) or (5*c*), respectively, which exactly satisfy the Fourier-space constraints. For the analysis of structure-factor trajectories generated by the RRR algorithm (Section 6) we followed the solution estimate given by equation (4*b*), which exactly satisfies the real-space constraints.

#### 2.1.4. The relaxed averaged alternating reflections (RAAR) algorithm

The relaxed averaged alternating reflections (RAAR) algorithm is a widely used IPA which was originally developed to control the noise-sensitivity of some earlier algorithms (Luke, 2005 ▸). The update rule for the RAAR algorithm is given by
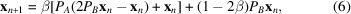
where β ∈ (0, 1) is an adjustable parameter. We note that for the special case of β = 1, the RAAR algorithm (equation 6) becomes equivalent to the revRRR algorithm with β = 1 (equation 5*a*[Disp-formula fd7]). Correspondingly, when β = 1 a solution estimate is readily calculated from the iterate using equations (5*b*) and (5*c*). However, when β ≠ 1 there is no obvious way to calculate the solution from the iterate, which represents a limitation of the algorithm. In a practical implementation of the RAAR algorithm, the value of β could be gradually increased towards 1 as the iterations proceed (Luke, 2005 ▸), which obviates this problem. For our purposes, we keep β fixed, as we do for the other algorithms, but we calculate a solution estimate using equation (5*c*), even when β ≠ 1, and use this to monitor agreement with the known solution.

Finally, we note that like the RRR algorithm, the RAAR algorithm is not symmetric, so that it is possible to generate a different algorithm by interchanging the projections in the update rule. However, we do not consider the ‘reversed’ RAAR algorithm.

### 2.2. Constraints and projections onto the constraints

The constraints in Fourier space are the measured Fourier amplitudes. Projection onto the constraints involves Fourier transforming the current density, replacing the Fourier amplitudes with the measured Fourier amplitudes and transforming back to real space. For unmeasured Fourier amplitudes, which are not subject to any direct constraint, statistical restraints are implemented based on Wilson statistics (Kingston & Millane, 2022 ▸), which prevent these terms taking on physically unrealistic values.

The constraints employed in real space are solvent flatness (the solvent region should be effectively featureless) and histogram equivalence (the protein region should have a characteristic density-value distribution). This amounts to assuming and enforcing priors for the density distribution in both the solvent and the protein region (where the prior in the solvent region is a one-point distribution). Application of the real-space constraints requires determination of the molecular envelope, a binary-valued function indicating which regions of the map are protein and which are solvent. As in our prior work (Kingston & Millane, 2022 ▸), the molecular envelope is updated on each iteration, based on thresholding the local variance of the solution estimate (Abrahams & Leslie, 1996 ▸; Terwilliger & Berendzen, 1999 ▸). Given an envelope, a projection onto the constraints involves setting the current density in the solvent region equal to its mean value, while applying an order-preserving transformation of the density values in the protein region that generates the desired histogram (Harrison, 1988 ▸; Lunin & Vernoslova, 1991 ▸). These changes are distance-minimizing (Elser, 2003 ▸). Further details regarding specification of the priors are given in Kingston & Millane (2022 ▸).

## 3. Other computational methods

### 3.1. Simulation strategy

Our initial objective was to investigate the suitability of a number of IPAs for crystallographic phase retrieval by exploring their convergence behavior as a function of their configurable parameter. We did this by randomly corrupting the density functions of previously determined protein crystal structures and testing the ability of the algorithms to return to the solution as the random error was increased. The advantage of this approach is that it allows algorithm convergence to be studied under well controlled conditions using a limited number of iterations, making the investigation computationally tractable. As we show (Sections 5.1 and 5.2), these experiments clearly identify the productive and unproductive regions of the parameter space for each algorithm studied.

A limitation of this approach is that it does not directly investigate performance of the algorithms for *ab initio* phase determination, beginning with completely randomized phase sets. In these circumstances, convergence to the solution may sometimes require many thousands of iterations (Kingston & Millane, 2022 ▸). A feature of *ab initio* phase determination is that coalescence of a near-correct molecular envelope (which is determined from the density estimate; Section 2.2) sometimes precedes convergence to the correct density by some margin, and hence appears to be a necessary ‘pre-step’ during direct phase determination. It is this observation which underpins our previous development of a two-stage procedure for direct phase determination, with the first stage involving formation of an approximation to the molecular envelope (Kingston & Millane, 2022 ▸). Some experiments that test the ability of the algorithms to perform direct phase determination, starting with random phases and an approximate molecular envelope, are reported in Section 5.3.

### 3.2. Error model

To introduce error into the density function, we manipulated the phases in the Fourier domain. For the acentric data, where there are no restrictions on the phase value, the phases calculated from deposited atomic models (φ_m_) were replaced with a von Mises distributed random variate φ (Fisher, 1993 ▸; Mardia & Jupp, 1999 ▸; Barnett & Kingston, 2024 ▸), with location parameter μ = φ_m_, defining the mean and mode of the distribution, and concentration parameter κ, defining its dispersion around the mean. Hence, the probability density function for φ is given by
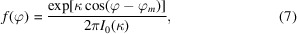
where *I*_0_ is the modified Bessel function of the first kind and order zero. The circular variance of the von Mises distribution is given by
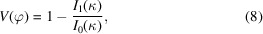
where *I*_1_ is the modified Bessel function of the first kind and order one. Computationally, a von Mises random variate was generated from a sequence of uniform random variates using the procedure of Best & Fisher (1979 ▸).

For the centric data, where there are only two possible phase values, the phases calculated from the deposited atomic models (φ_m_) were replaced with a wrapped Bernoulli distributed random variate φ with probabilities *p* (associated with the model phase φ_m_) and *q* = 1 − *p* (associated with phase φ_m_ + π). Setting *p* = 1 introduces no phase error, setting *p* = 0.5 fully randomizes the centric phases and setting *p* = 0 exactly switches all centric phases. The probability mass function for φ is given by
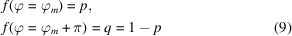
and the circular variance of the wrapped Bernoulli function is given by (Girija *et al.*, 2014 ▸)
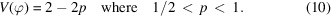


The probability *p* was set such that the circular variance of the wrapped Bernoulli function (equation 10) and the von Mises distribution (equation 8) were equal.

We note that for any specified circular variance, the same error distributions were used for all centric and acentric data. A more sophisticated model might scale the error according to the amplitude or frequency of the Fourier terms, as the large-amplitude terms contribute more to the variance of the density function than the small terms (Giacovazzo & Mazzone, 2011 ▸; Giacovazzo *et al.*, 2011 ▸) and are also more critical to the convergence of conventional iterative density-modification procedures (Vekhter, 2005 ▸; Uervirojnangkoorn *et al.*, 2013 ▸). However, the simple error model is sufficient for our purpose.

### 3.3. Global agreement measures

To monitor the agreement between phase sets and density functions, several metrics were employed.

The mean absolute phase difference (mean unsigned phase difference) was used as a simple measure of phase dispersion,
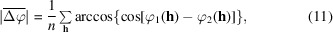
where φ_1_(**h**) and φ_2_(**h**) are the phase sets being compared, *n* is the number of terms in the summation and the trigonometric functions act to place the phase difference in the domain 0 < Δφ < π.

The Pearson correlation coefficient was used as a measure of real-space agreement between two density functions. This is  conveniently calculated from the Fourier amplitudes *F*_1_(**h**) and *F*_2_(**h**) and phase differences Δφ(**h**) = Δφ_1_(**h**) − Δφ_2_(**h**) (Lunin & Woolfson, 1993 ▸; Bailey *et al.*, 2012 ▸) using
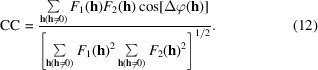


Finally, as a measure of the correlation between two phase sets, the circular correlation coefficient defined by Fisher & Lee (1983 ▸) was employed, which in this setting can be written as
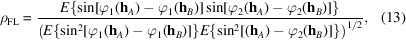
where **h**_*A*_ and **h**_*B*_ are the indices of any two observations in the data set and *E* is the expected value. Similar to the linear correlation coefficient, ρ_FL_ takes on values between −1 and 1, with +1 indicating positive association between the phase sets and −1 indicating negative association. If the two phase sets are independent then ρ_FL_ = 0. We note that there are many alternate definitions of correlation for angular variables (Jupp & Mardia, 1989 ▸).

As an estimator of ρ_FL_ we evaluate (Fisher, 1993 ▸)
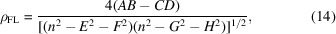
where *n* is the number of observations and
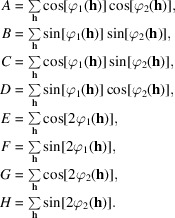


Equations (11), (12) and (14) are correct where the summations take place over the full hemisphere of data in reciprocal space. In summations that extend only over the asymmetric unit, the terms must be weighted by statistical factors ɛ(**h**), which account for the variable degeneracy of the reciprocal-lattice points (Blessing *et al.*, 1998 ▸; Iwasaki & Ito, 1977 ▸).

### 3.4. Analysis of structure-factor trajectories

In Section 6, we analyze structure factors generated by the RRR algorithm as a function of iteration (*i.e.* structure-factor trajectories). To aid the visualization of these trajectories, the structure factors for acentric data were modeled as independently Gaussian-distributed on amplitude (*F*) and von Mises-distributed on phase (φ), with probability density functions
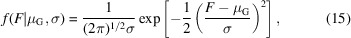

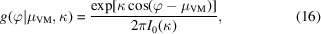
where the Gaussian distribution is characterized by its mean (μ_G_) and variance (σ) and the von Mises distribution is characterized by its location (μ_VM_) and concentration (κ) parameters.

Under the assumption of independence, the joint PDF of the Fourier coefficients is then
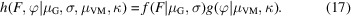


The estimators of the Gaussian distribution parameters μ_G_ and σ were the sample mean and sample standard deviation of the amplitudes over the relevant region of the trajectory, respectively. To obtain estimators of the von Mises distribution parameters μ_VM_ and κ, we first computed the sample mean length (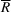) and sample mean direction (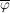) of the phases according to Fisher (1993 ▸) and Mardia & Jupp (1999 ▸),
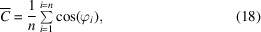

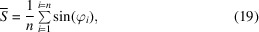

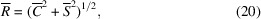

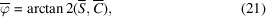
where φ_*i*_ are the phase estimates over the relevant region of the trajectory and arctan2 denotes the four-quadrant inverse tangent.

The maximum-likelihood estimator of the von Mises location parameter μ_VM_ is simply the sample mean direction (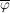). The maximum-likelihood estimator of the von Mises concentration parameter κ is given by the solution of (Fisher, 1993 ▸; Mardia & Jupp, 1999 ▸)
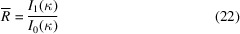
for κ, which was evaluated using the algorithm of Hill (1981 ▸).

For nonparametric analysis of phase-angle distributions following convergence (Section 6.2), we simply computed the sample mean length (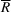) and sample mean direction (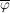) from the phase-angle trajectory in the stationary region for both centric and acentric structure factors using equations (18)–(21). In Supplementary Fig. S3 and Supplementary Movie S1 we report some results in terms of the sample circular variance (1 − 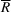).

To compute the phase-retrieval transfer function (Chapman *et al.*, 2006 ▸) following convergence (Section 6.3) we evaluated
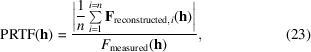
where **F**_reconstructed,*i*_(**h**) are the complex-valued Fourier co­efficients generated by an IPA in the stationary part of its trajectory and *F*_measured_(**h**) are the experimentally measured Fourier amplitudes. We then calculated the mean of the statistic in equation (23) as a function of resolution (*i.e.* averaged over concentric spherical shells in Fourier space), correcting appropriately for the variable degeneracy of the lattice points in the asymmetric unit (Blessing *et al.*, 1998 ▸; Iwasaki & Ito, 1977 ▸).

### 3.5. Test cases

In the figures we present results generated using several crystallographic data sets, which are summarized in Table 1 ▸. All of these diffraction data were collected from crystals with a solvent content exceeding 60%, creating a strong constraint on the density function. PDB entries 4bsj and 4zqk are used for the comparative experiments described in Sections 5.1 and 5.2. PDB entry 4bsj is used for the illustrative analysis in Section 6.1. PDB entries 4bsj and 4gbg are used to demonstrate the effects of averaging over the stationary part of the algorithm trajectory (Section 6.2). PDB entry 4bsj is used to demonstrate the behavior of the phase-retrieval transfer function (Section 6.3). PDB entry 4nli is used to make some supplementary points about the behavior of the RRR algorithm as the solution is located (Supplementary Movie S1) and the response of the structure-factor distributions to the configurable algorithm parameter β (Supplementary Fig. S3).

Finally, PDB entry 4bsj, together with four additional test cases, is used to demonstrate the effectiveness of the algorithms for *ab initio* phase retrieval in Section 5.3. Details are given in Supplementary Table S1.

### 3.6. Implementation

All of the algorithms described in the paper (Section 2.1) have been implemented within the program *IPA* (version 1.2), which is available on Github (https://github.com/rlkingston/IPA). All other computational procedures required to replicate the results are now accessible to the user, including the ability to introduce controlled amounts of phase error using circular probability distributions and the ability to average over the stationary part of the algorithm trajectory and evaluate the phase-retrieval transfer function (equation 23).

## 4. The error model and its relationship to phase and electron-density agreement

To enable simulations, density functions were perturbed by introducing phase error into the structure factors using appropriate circular probability distributions, as described in Section 3.2. The error distributions were parameterized so that they had a defined circular variance. Here, we establish the relationship between the circular variance of the phase-error distributions and the statistics given by equations (11–13) used to monitor phase and electron-density agreement.

**Figure 1:**
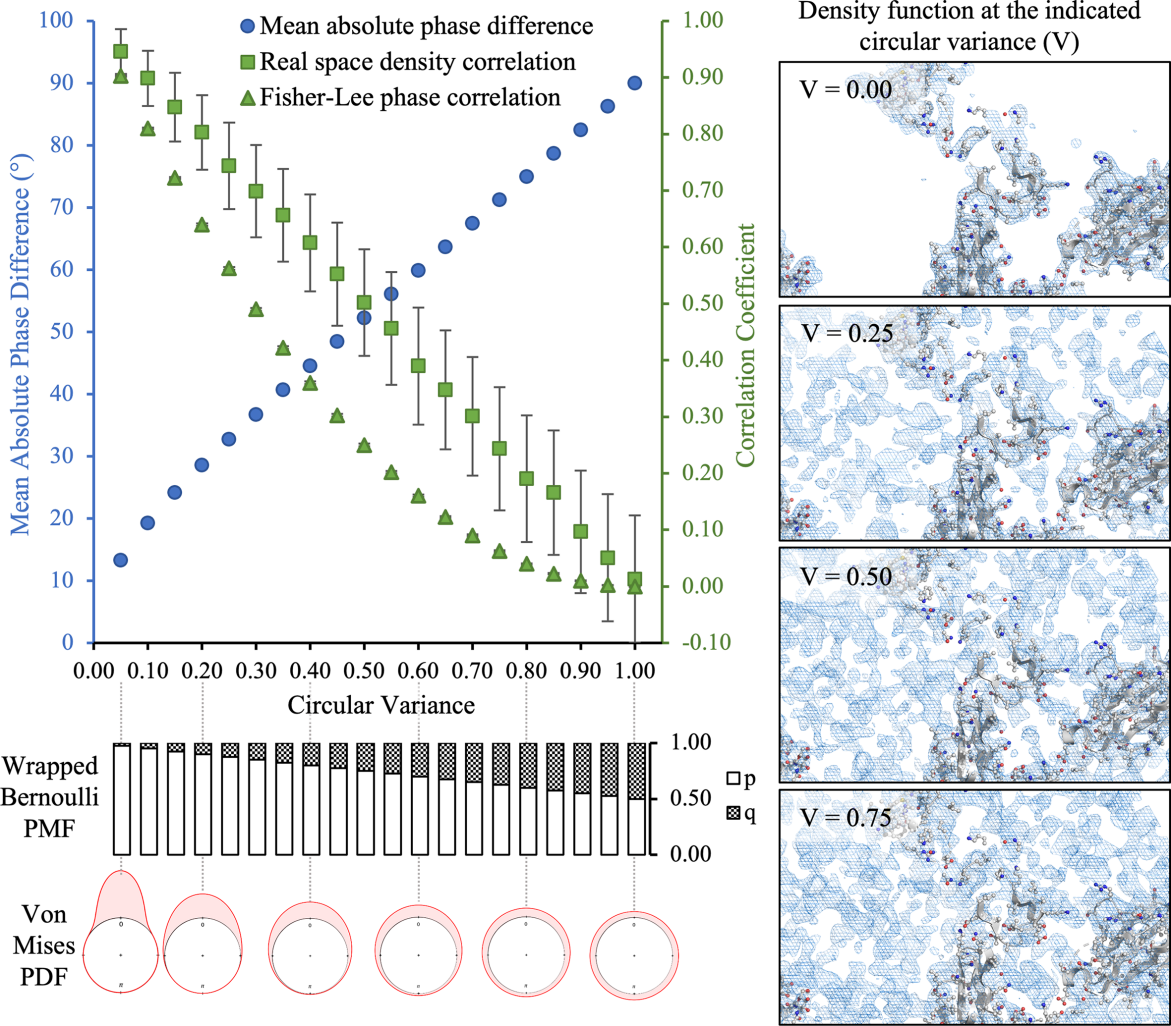
Density and phase agreement measures as a function of the circular variance of the phase-error distributions. The top left panel shows the Fourier-space mean absolute phase difference (equation 11), the real-space density correlation coefficient (equation 12) and the Fourier-space Fisher–Lee phase correlation coefficient (equation 13) as a function of the circular variance of the phase-error distributions for test case 4bsj. Symbols show the sample mean for each statistic, while error bars show half the sample standard deviation. The bottom left panel shows schematically the correspondent probability mass function of the wrapped Bernoulli function, used to introduce phase error for the centric data, and the probability density function of the von Mises distribution, used to introduce phase error for the acentric data. The von Mises distribution is displayed on the unit circle, with the distance from the unit circle at each angle representing the probability density, and the location parameter μ set to π/2, without loss of generality. The insets on the right show the typical appearance of the electron-density function at the indicated level of error, with the same isosurface displayed in all cases.

For each circular variance, 1000 phase sets were generated with random errors incorporated and agreement statistics were calculated with the original data set. Some typical results are shown in Fig. 1 ▸ (for PDB entry 4bsj). Both the mean absolute phase difference (equation 11) and the real-space density correlation (equation 12) are almost linear functions of the circular variance, while the Fisher–Lee phase correlation (equation 13) responds in a less linear fashion. Of the three agreement metrics, only the real-space density correlation shows appreciable variation around its mean value, with the magnitude of this variation differing between crystallo­graphic data sets (data not shown). This reflects the appearance of the Fourier amplitudes in the summation used to calculate the statistic (equation 12).

## 5. Behavior of IPAs as a function of their adjustable algorithm parameter

For the phase-retrieval problem, the initial objective was to explore how iterative projection algorithms behave, when initiated at a varying distance from the solution, as a function of their adjustable algorithm parameter. In other words, if we move some defined distance from the solution by randomly perturbing the phases, what is the ability of the algorithms to return to the solution?

### 5.1. Behavior of the DM algorithm when initiated at a varying distance from the solution

Our initial investigations of this question used the DM algorithm (equation 3*a*[Disp-formula fd3]), which we have previously shown to be effective for *ab initio* phase determination when the constraints on the image are sufficiently strong (in particular, when the crystal solvent content is >70%; Kingston & Millane, 2022 ▸). The algorithm was deployed on two test cases (PDB entries 4bsj and 4zqk), representing phase-retrieval problems of varying difficulty. Test case 4bsj has a solvent fraction of 0.74. In this case *ab initio* phase determination is known to be possible, and hence the solution to the phase-retrieval problem must be uniquely determined by the available constraints (Kingston & Millane, 2022 ▸). Test case 4zqk has a solvent fraction of 0.61. In this case the feasibility of *ab initio* phase determination has not been demonstrated (Kingston & Millane, 2022 ▸).

The trajectory of the iterate in an IPA can vary dramatically and unpredictably for a particular problem, depending on the initial state. This reflects the difficulty of the phase-retrieval problem: the algorithms are being used to explore a high-dimensional space, subject to nonconvex constraints. Even when the solution is uniquely specified by the constraints, the number of iterations required to locate the solution can vary widely, depending on the starting state, and the solution may not be located within a computationally reasonable number of iterations. Analyzing algorithm behavior therefore requires extensive replication with different randomly generated initial states to characterize the statistical distribution of the results.

Consequently, the experimental approach taken was as follows. For each of the two test cases, phase sets calculated from the deposited atomic models were corrupted with random error using the model described in Section 3.2. The circular variance (*V*) of the applied phase-error functions ranged from 0.1 to 0.9. The correspondent variation in phase and map agreement measures, and typical appearance of the resulting density function, are shown in Fig. 1 ▸, which illustrates how the circular variance of the error functions controls the fidelity of the density function. At each circular variance, 30 random replicates were generated and used as input to the DM algorithm (executed for a fixed 250 iterations and with adjustable parameter β = 0.75). Although it is known that gradually increasing the resolution of the density function, through the application of a Fourier-space data-apodization scheme, aids the convergence of iterative phase-retrieval algorithms (Lo *et al.*, 2015 ▸; He & Su, 2018 ▸; Kingston & Millane, 2022 ▸), the calculations here were carried out without apodization for simplicity.

**Figure 2:**
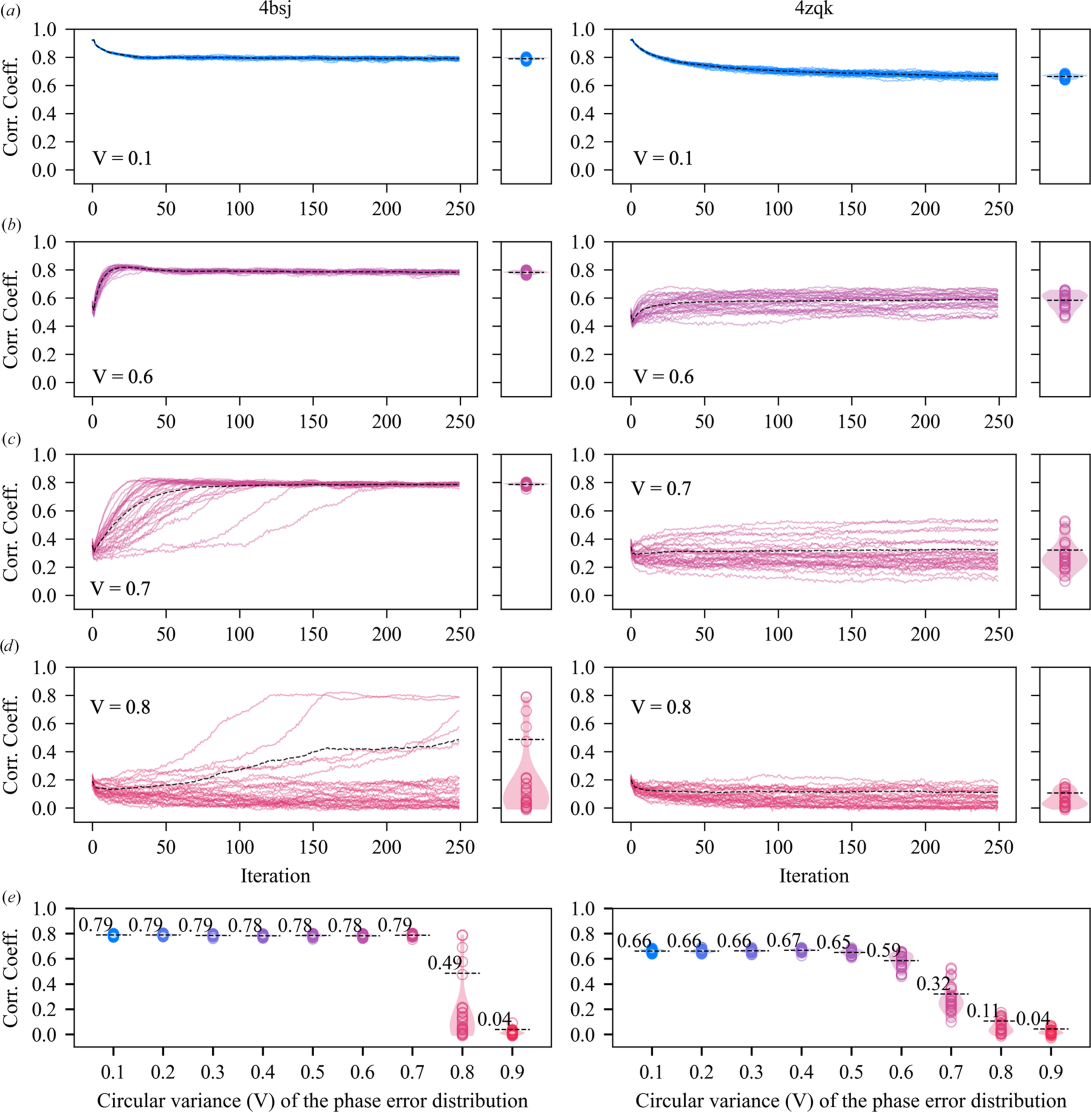
Behavior of the difference-map algorithm (β = 0.75) starting at a varying distance from the solution for test cases 4bsj (left panels) and 4zqk (right panels). (*a*)–(*d*) show trajectories of individual replicates, with initial error specified by the circular variance (*V*) of the phase-error distribution as indicated. The real-space correlation coefficient with the known solution is displayed as a function of iteration. The weighted average trajectory, evaluated from all replicates, is shown with a dashed black line [each trajectory being self-weighted (Garcia, 2012 ▸) according to the value of the correlation coefficient at each iteration]. The associated violin plots (Hintze & Nelson, 1998 ▸) summarize the agreement of the individual replicates at the final iteration. (*e*) summarizes the overall results of the experiment, showing the violin plots as a function of the circular variance of the phase-error distributions.

The results of the experiment are shown in Fig. 2 ▸. The real-space correlation coefficient with the original density function is used as the measure of agreement throughout. The trajectories of individual replicates, at varying error levels (defined by the circular variance of the phase-error distributions), are shown in Figs. 2 ▸(*a*)– ▸(*d*) together with violin plots (Hintze & Nelson, 1998 ▸) that summarize the agreement at the end of the runs. The overall results of the experiment are shown in Fig. 2 ▸(*e*).

With small amounts of added error (*V* ≤ 0.5 or 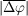 ≤ 50°) all runs rapidly converge close to the known solution (Fig. 2 ▸*e*). The final mean map correlations are ∼0.78 for test case 4bsj and ∼0.65 for test case 4zqk. In this regime, the point of convergence remains essentially the same, although the algorithm may be initiated at a point that is closer to the known solution (Fig. 2 ▸*a*) or more distant from the known solution (Fig. 2 ▸*b*). This suggests that the results at low error simply reflect the ability of the constraints (solvent flatness and histogram equivalence) to maintain the image at a point close to the solution. Those constraints are considerably less powerful for test case 4zqk (solvent fraction 0.61) than for test case 4bsj (solvent fraction 0.74), although we note that even among test cases with equivalent solvent content there is considerable variation in the ability of the algorithms to maintain the solution (data not shown).

As the error levels increase (*V* > 0.5 or 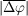 > 50°), and the problem begins to resemble *ab initio* phase retrieval, the proportion of runs which return to the solution in 250 iterations declines. Differences in the behavior of test cases 4bsj and 4zqk become apparent. For 4bsj, the lag before the solution is located increases with the error level, but the algorithm remains capable of locating the solution in 250 iterations when *V* = 0.8 (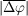 ≃ 75°; Fig. 2 ▸*d*). In this case the runs which progress to intermediate values of agreement in 250 iterations (Fig. 2 ▸*d*) would converge to the solution if more iterations were allowed (Supplementary Fig. S1 shows a replicate of the experiment in which 1000 iterations are completed). For 4zqk the phase-retrieval process is less robust to added error, and there is no indication of progression towards the solution at *V* = 0.8, whatever the number of iterations (Fig. 2 ▸*d*, Supplementary Fig. S1). This is consistent with the weaker constraints being insufficient for *ab initio* phase retrieval. Overall, in the higher error cases, the final result clearly reflects both the power of the constraints and the efficiency of the iterative projection algorithm in locating the solution in a fixed number of iterations.

With very large amounts of added error (*V* = 0.9 or 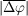 ≃ 85°) the initial phases are effectively random, and no runs return to the solution for either test case in 250 iterations, although the constraints are sufficiently powerful to directly determine the solution for test case 4bsj if more iterations were allowed (Kingston & Millane, 2022 ▸).

As a control we performed the same basic experiment using the ER (Gerchberg–Saxton) algorithm (equation 2). The results are shown in Supplementary Fig. S2. Consistent with its known properties (Stark & Yang, 1998 ▸), the ER algorithm is effective when initiated close to the solution, but is much less effective than the DM algorithm when initiated far from the solution. At high error (*V* > 0.7 or 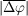 > 70°) the ER algorithm never appears to progress significantly from the point at which it is initiated for either test case, when following a global agreement measure such as the density correlation. At low error, the ER algorithm does produce much better agreement with the solution than the DM algorithm. However, we show in Section 6.2 how this apparent loss of accuracy when using the DM algorithm could readily be rectified.

### 5.2. Effectiveness of various IPAs as a function of their adjustable parameter β

The DM algorithm (equation 3*a*[Disp-formula fd3]) is one of a number of IPAs that have been used to solve difficult nonconvex constraint-satisfaction problems. We explored the utility of several other algorithms for iterative phase retrieval that have seen little application in crystallography to date. These are the RRR algorithm (equation 4*a*[Disp-formula fd5]), a reversed variant of the RRR algorithm (equation 5*a*[Disp-formula fd7]) and the RAAR algorithm (equation 6), which are described in Section 2.1. We note that the RAAR algorithm has previously been employed to determine the anomalous scattering substructure from single-wavelength anomalous diffraction data (Skubák, 2018 ▸; Fu *et al.*, 2024 ▸).

Like the DM algorithm, the behavior of each of these algorithms is dependent on a single adjustable parameter β, although the range of β differs between the algorithms. As the optimal choice of β is domain-specific, we systemically investigated the effects of the parameter β on the performance of the algorithms for crystallographic phase retrieval.

**Figure 3:**
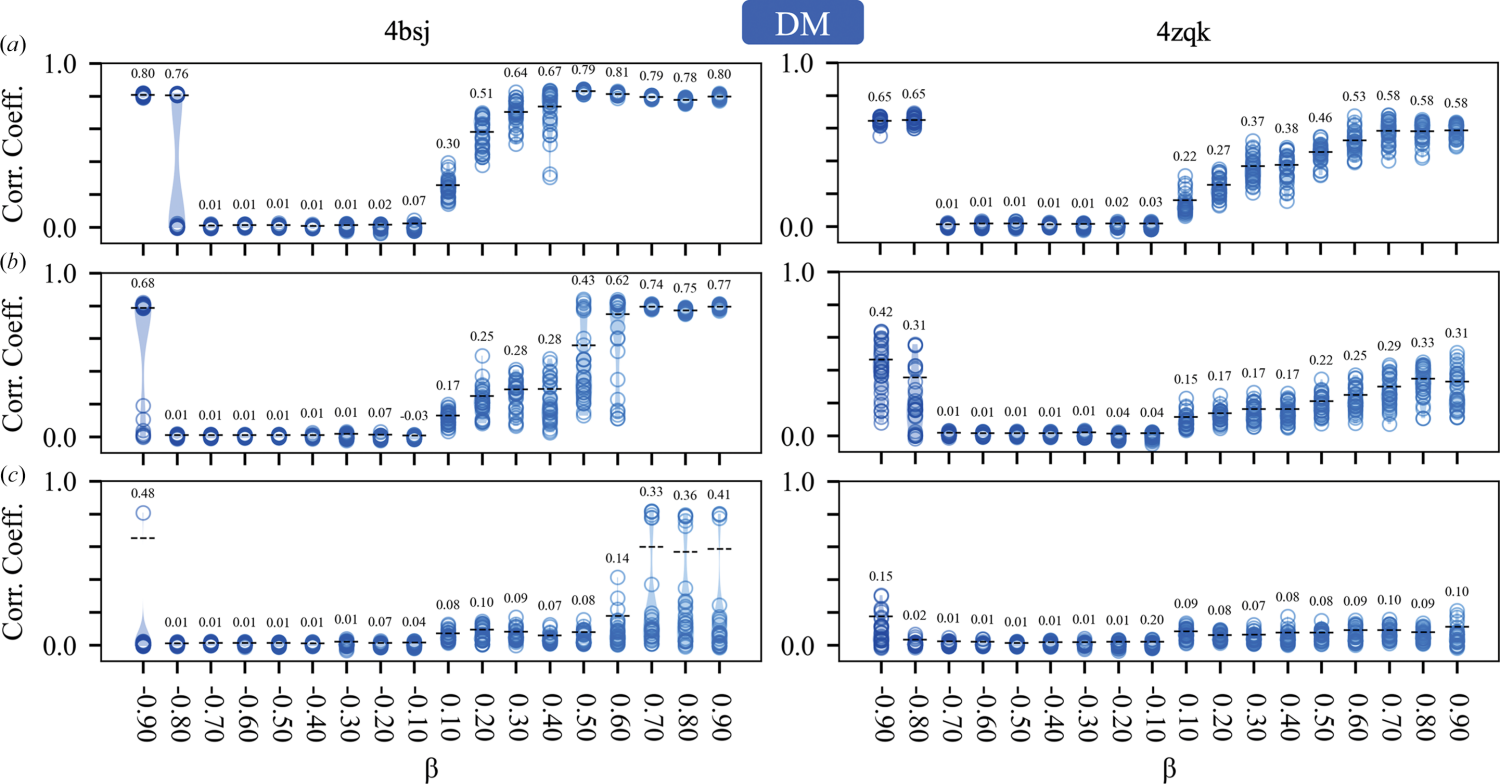
Performance of the DM algorithm as a function of its adjustable parameter β for test cases 4bsj and 4zqk. Violin plots (Hintze & Nelson, 1998 ▸) summarize the agreement (real-space correlation coefficient) of the individual replicates with the known solution at the final iterate. As in Fig. 2 ▸, the black dashed line indicates the self-weighted (Garcia, 2012 ▸) mean correlation coefficient calculated from the replicates. (*a*) Results for error-function circular variance *V* = 0.6 (mean absolute phase difference 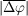 = 60°, starting map correlation coefficient ∼0.39). (*b*) Results for error-function circular variance *V* = 0.7 (mean absolute phase difference 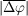 = 68°, starting map correlation coefficient ∼0.30). (*c*) Results for error-function circular variance *V* = 0.8 (mean absolute phase difference 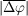 = 75°, starting map correlation coefficient ∼0.19).

Our initial experiments (Section 5.1) established that the failure point of the DM algorithm (with β = 0.75 and a fixed 250 iterations) occurred when the circular variance of the error functions was 0.7–0.8, depending on the test case (Fig. 2 ▸*e*). This corresponds to mean absolute phase differences of 70–75° and starting map correlations of 0.3–0.2 with the known solution (Fig. 1 ▸). With higher levels of phase error, the DM algorithm was unable to routinely recover the solution for either test case within 250 iterations. Based on these results, we investigated the ability of all of the algorithms to recover the solution, as a function of their parameter β, given similar levels of initial error (*V* = 0.6–0.8) and the same number of iterations (250). The results are summarized in Figs. 3 ▸, 4 ▸, 5 ▸ and 6 ▸, which show agreement with the known solution at the final iteration.

For the DM algorithm, where β ∈ (−1, 1), the results are consistent with our earlier empirical observations (Kingston & Millane, 2022 ▸). The effectiveness of the algorithm is quite sensitive to the value of β, and it works most robustly when β ≥ 0.7 or β < −0.9 (Fig. 3 ▸). While adopting negative values for β amounts to swapping the order in which the projections are applied within the update rule (equation 3*a*[Disp-formula fd3]), the response to β is not symmetric, and performance is more sensitive to the exact value of β in the negative region. There also exists a range of values −0.7 < β < 0.1 which are entirely unproductive. We note that terms involving 1/β appear in the update rule of the DM algorithm (equation 3*a*[Disp-formula fd3]), so irrespective of our results the algorithm is not defined when β = 0. The data further suggest that the useful values for β are somewhat dependent on the level of error in the density function. For example, β = 0.5 works well at relatively low error (Fig. 3 ▸*a*) but becomes much less effective at recovering the solution at high error (Fig. 3 ▸*c*). There is of course no reason why β must be held constant during the phase-retrieval process and we have previously found, empirically, that varying β as a function of the iterate can aid in convergence of the DM algorithm (Kingston & Millane, 2022 ▸).

**Figure 4:**
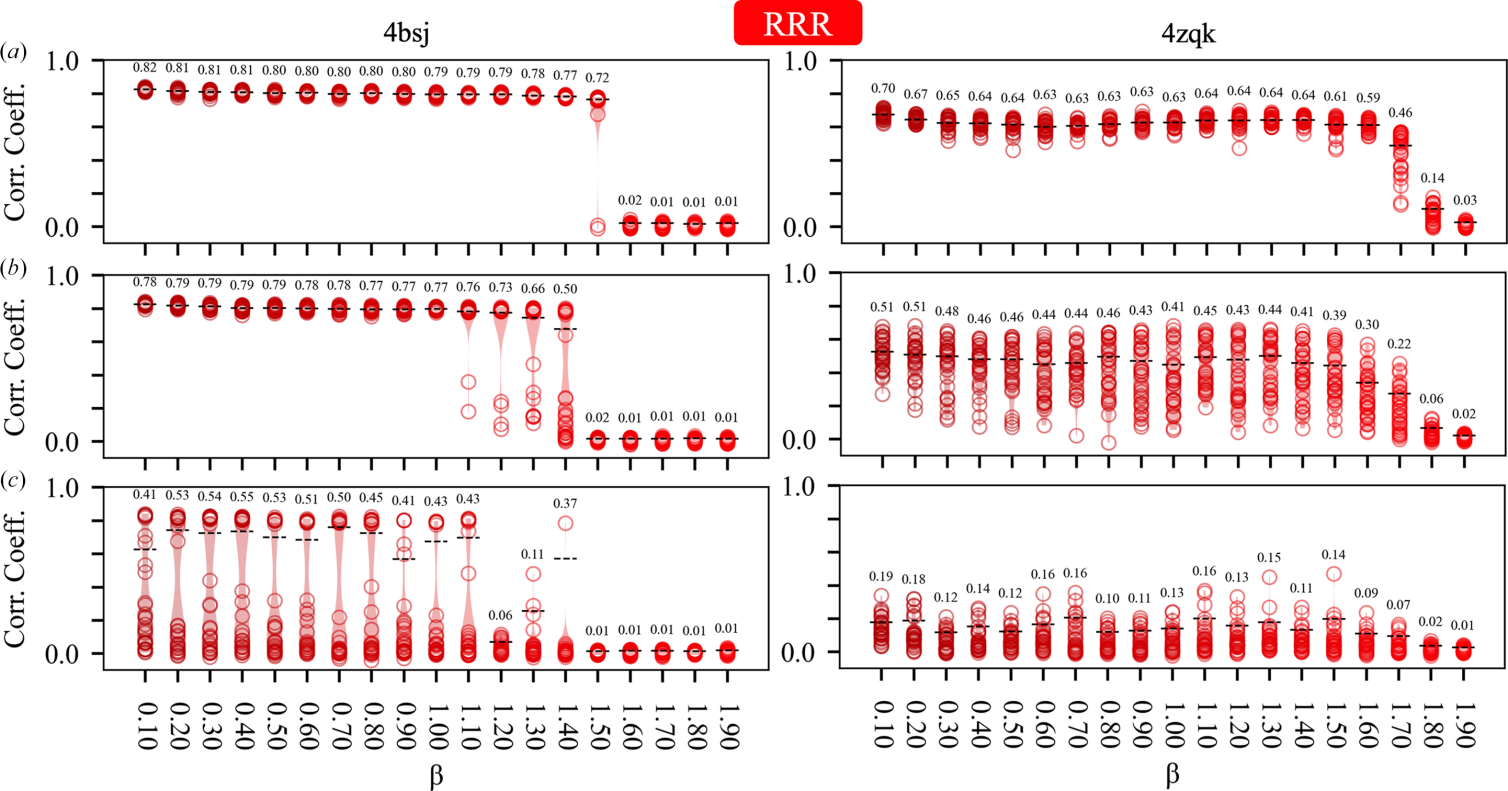
Performance of the RRR algorithm as a function of its adjustable parameter β for test cases 4bsj and 4zqk. Details are as for Fig. 3 ▸.

The RRR algorithm and the revRRR algorithms, where β ∈ (0, 2), exhibit a quite similar response to β, with 0.2 < β < 1.2 being the most productive values for phase retrieval and β > 1.7 being generally unproductive. As with the DM algorithm some sensitivity to the level of error is apparent, with borderline values, such as β = 1.5, being effective at low error and ineffective at high error (test case 4bsj; Figs. 4 ▸ and 5 ▸). However, overall, the RRR and revRRR algorithms show less sensitivity to the exact value of β than the DM algorithm.

The RAAR algorithm, where β ∈ (0, 1), exhibits a less step-like response to β than either the DM or RRR algorithms. Overall, it appears that the region with β > 0.75 is the most productive and the region with β ≤ 1/2 is less productive, which is not unexpected. In the limit, when β = 0, the update rule for the RAAR algorithm (equation 6) does not involve any projection onto the real-space constraints. Our findings are consistent with prior investigation of the performance of the RAAR algorithm in noncrystallographic phase-retrieval problems (Li & Zhou, 2017 ▸).

### 5.3. Effectiveness of various IPAs for direct phase retrieval

Following the basic characterization of algorithm behavior, we tested the ability of the algorithms to perform true *ab initio* phase retrieval. In these experiments we used each algorithm (DM, RRR, revRRR and RAAR, with ‘optimal’ choices for β) to directly phase five different test cases beginning with completely random phases and algorithmically determined approximations to the molecular envelope. The test cases employed (Supplementary Table S1) were a subset of those used in Kingston & Millane (2022 ▸). These phase-retrieval experiments involved many more iterations (8000) than the experiments shown in Figs. 3 ▸, 4 ▸, 5 ▸ and 6 ▸ and graduated extension of the resolution via a Fourier-space apodization scheme (Kingston & Millane, 2022 ▸). The results (Supplementary Table S1) establish that all algorithms are effective for direct phase retrieval, although there is considerable case-by-case variation in algorithm performance, which remains to be explored.

## 6. Analysis of algorithm behavior in the Fourier domain

### 6.1. Monitoring individual structure-factor trajectories as the algorithms progress

The experiments described in the previous section probed the global performance of IPAs as a function of their adjustable parameter, and established their utility for direct phase determination. Additional insight into algorithm behavior can be obtained by examining the trajectories of individual Fourier coefficients as the algorithms progress. This concept is illustrated here using the RRR algorithm alone, as the behavior of the other algorithms studied is broadly similar.

**Figure 7:**
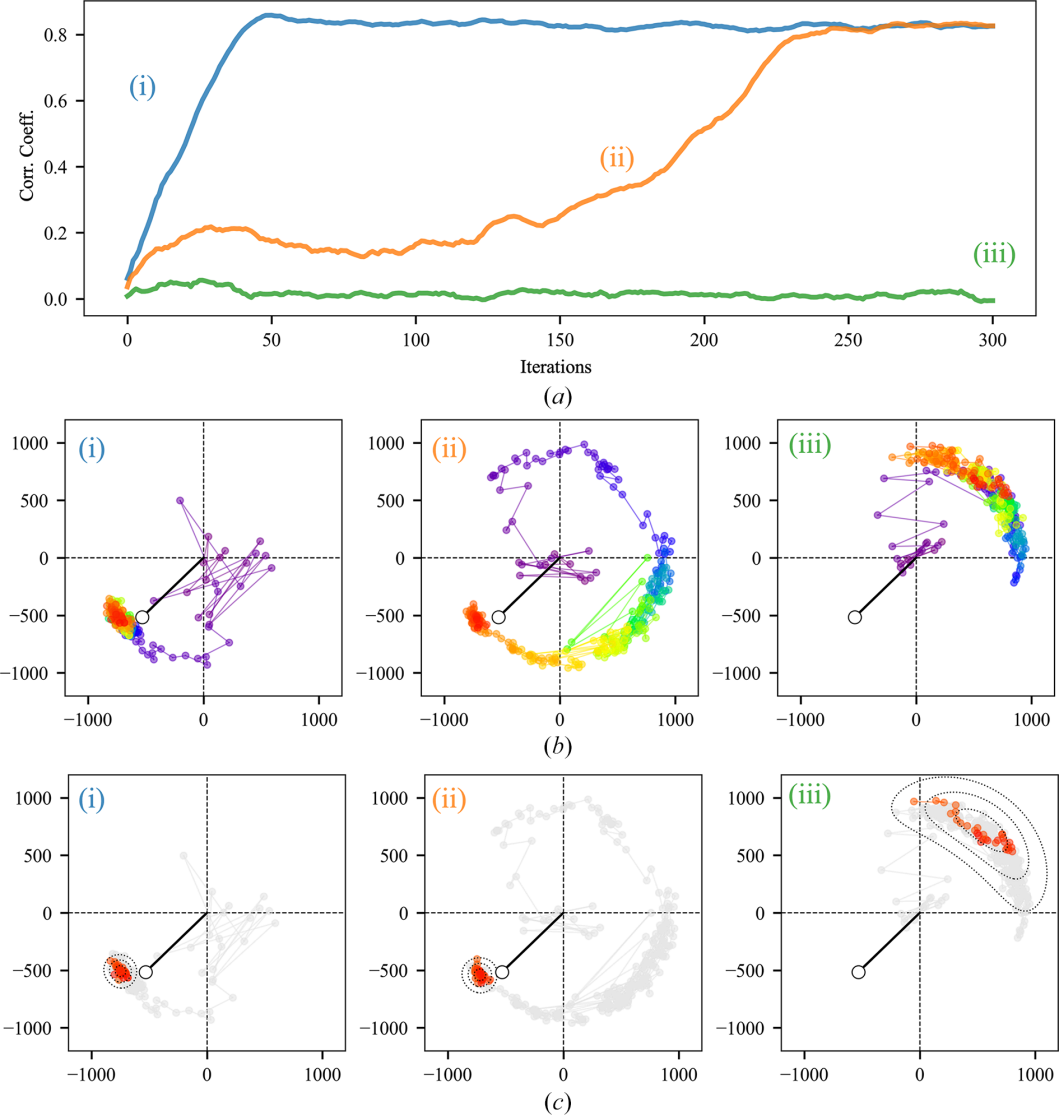
Global agreement statistics and individual structure-factor trajectories generated by the RRR algorithm (β = 0.80, test case 4bsj) following initiation of the algorithm with three different random phase sets (i), (ii) and (iii). For phase sets (i) and (ii) the algorithm converges to the global solution within 300 iterations, while for set (iii) it does not. (*a*) Evolution of the real-space map correlation coefficient, an overall agreement measure. (*b*) Trajectories for the Fourier coefficient with indices *h* = 12, *k* = 9, *l* = 8 for the three runs. The estimate for the Fourier coefficient at each iterate, obtained from equation (4*b*), is represented with a filled circle in the complex plane, and consecutive iterates are connected with thin lines. The progression of the trajectory over the 300 iterations allowed is indicated with a purple-to-red color gradient. The experimentally measured structure-factor amplitude at the model-derived phase angle is indicated by an open circle, connected to the origin by a thick black line. (*c*) Fit of the final 30 iterations in the algorithm trajectory to a bivariate probability density function, assuming independent von Mises distribution of the phases and Gaussian distribution of the amplitudes (equation 17). Points contributing to the fit of the PDF retain their color, while all remaining points in the trajectory are reverted to gray. The displayed isocontours of the fitted PDF pass through μ_G_ ± 2σ, μ_G_ ± 4σ and μ_G_ ± 6σ along the central symmetry axis of the distribution.

Phase retrieval was conducted for test case 4bsj using the RRR algorithm (β = 0.80 and 300 iterations) initiated with three different random phase sets, and the results are shown in Fig. 7 ▸. The known molecular envelope was used at iteration 0, and the envelope was updated based on the density function at each iteration thereafter. The use of a correct or near-correct molecular envelope to initiate phase retrieval has the effect of decreasing the mean number of iterations required for convergence, as we have previously noted (Kingston & Millane, 2022 ▸) and exploited in Section 5.3. For two of the phase sets the algorithm converges to the global solution, while for the third phase set it does not, as indicated by the global agreement statistics (Fig. 7 ▸*a*).

Trajectories of a single Fourier coefficient generated by the RRR algorithm (test case 4bsj) over the course of each run are shown in Fig. 7 ▸(*b*). The experimentally measured amplitude and model-derived phase are represented by the thick black line terminating on an open circle. In each trajectory, variations in both amplitude and phase are apparent, as the algorithm attempts to find an intersection between the real- and Fourier-space constraints. Although the measured amplitudes act as the Fourier-space constraint on every iteration of the RRR algorithm, the trajectory of the solution estimate (equation 4*b*[Disp-formula fd6]) is being followed in Fig. 7 ▸(*b*). For this solution estimate, the real-space constraints (solvent flatness and histogram equivalence) are exactly satisfied on every iteration; however, the Fourier-space constraints are not, even at the solution (due to both errors in the measured amplitudes and the approximations inherent in the real-space constraints).

In the cases that converge (i and ii), the structure-factor trajectory eventually becomes stationary, with a mean value that does not change with iteration, consistent with the construction of the update rule for the iterate (equation 4*a*[Disp-formula fd5]). In this regime, the structure factor is undergoing what resembles a biased random walk in the complex plane and the distribution of estimates generated by the algorithm appears to be unimodal and symmetric. Consequently, for the purposes of visualization, we fit the final points in the structure-factor trajectory to a probability density function (Fig. 7 ▸*c*), modeling the phases as von Mises distributed, and the amplitudes as Gaussian distributed, and assuming independence between these two components (equation 17). When the algorithm has converged, the average structure factor across the final points in the trajectory is very close to the model-associated value, although not exactly coincident (Fig. 7 ▸*c*), noting that we are not estimating or representing errors in the model-derived phases (Read, 1997 ▸) or the measured amplitudes.

In contrast, the structure-factor trajectory for the case that did not converge (iii) is markedly different. At the algorithm end point, the trajectory is clearly nonstationary. Concomitantly, the distribution of points is far broader, and the average does not even approximately correspond to the model-associated value (Fig. 7 ▸*c*). We note that since this case is well constrained, it is likely that this trajectory would ultimately converge to the correct solution given an increased number of iterations.

A notable feature of the structure-factor trajectories shown in Fig. 7 ▸ is that even following convergence to the solution, constant movements around the mean value are apparent. This is the Fourier-space corollary of the significant fluctuations that are observed in the electron-density function, subsequent to the formation of an essentially correct image.

These fluctuations are shown explicitly in Supplementary Movie S1 (for a different test case, PDB entry 4nli), in which the trajectory of the density function, the trajectory of several individual Fourier coefficients and the circular variance of the phase-angle distributions are displayed both prior and subsequent to location of the solution. Convergence to the solution is accompanied by a general reduction in the variance of the phase-angle distributions as the structure-factor trajectories become stationary. This is quite diagnostic of successful phase retrieval. Nonetheless, the variance remains far from zero, and continued significant movements around the mean position are seen in both the density function and the Fourier coefficients following convergence to the solution. These fluctuations are an inevitable consequence of the design of algorithms such as RRR and occur in practical situations, when the constraints cannot all be simultaneously and exactly satisfied.

**Figure 8:**
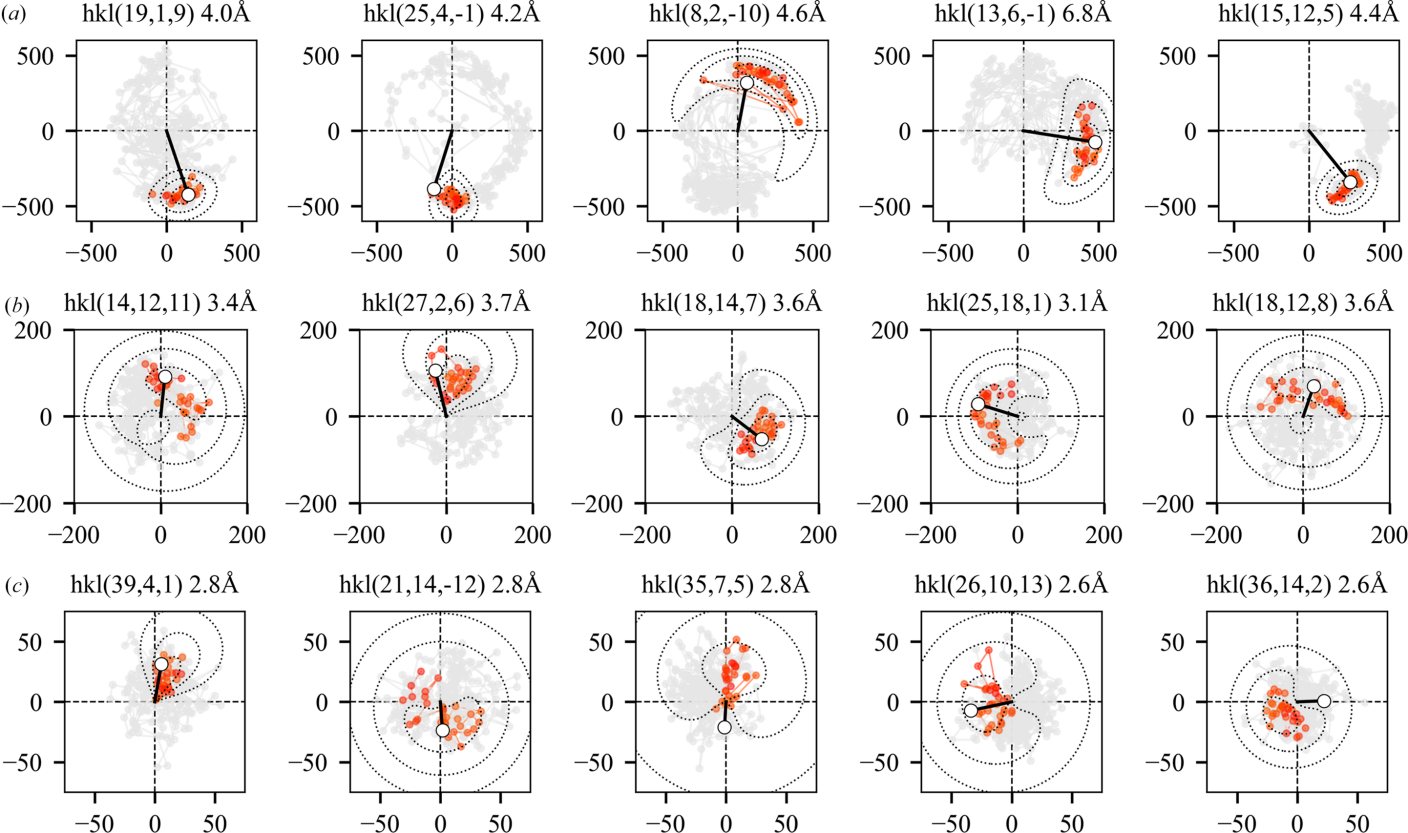
Structure-factor trajectories generated by the RRR algorithm (β = 0.8) following global convergence to the solution (test case 4bsj). Structure-factor trajectories for 15 individual acentric terms are displayed as in Fig. 7 ▸. Initial iterations of the trajectory are displayed in gray, while the final 30 iterations of each trajectory, which are representative of behavior following global convergence to the solution, are displayed in red. The trajectories following convergence (red points) are summarized via the fit of a bivariate probability density function. Isocontours of the PDF are displayed together with the model-derived structure factors, as in Fig. 7 ▸(*c*). (*a*) The large terms, displayed in the top row, fall in the 90th to 100th percentile of the measured amplitude distribution. (*b*) The intermediate terms, displayed in the middle row, fall in the 45th to 55th percentile of the measured amplitude distribution. (*c*) The small terms, displayed in the bottom row, fall in the 0th to 10th percentile of the measured amplitude distribution. Note that the scale for the amplitude is different for the large, intermediate and small terms to facilitate visualization.

To reinforce this point, we show in red in Fig. 8 ▸ the final trajectories of 15 individual acentric Fourier coefficients generated by one run of the RRR algorithm (β = 0.80) for test case 4bsj after convergence to the solution. The terms were selected based on the amplitude, having either large, intermediate or small values (top, middle and bottom rows, respectively). The trajectories of the large-amplitude Fourier terms are generally far more tightly constrained than the small-amplitude terms. This is unsurprising, as the large-amplitude terms will dominate the variance of the Fourier synthesis (Giacovazzo & Mazzone, 2011 ▸; Giacovazzo *et al.*, 2011 ▸) and will have the greatest impact on its appearance. However, among terms of nearly equivalent amplitude, there still exist considerable differences in the extent of the phase variation following convergence, implying that the phases for individual terms in the Fourier synthesis are not equally well determined by the constraints being applied. Despite the breadth of the distributions for the small- and intermediate-amplitude terms, they are generally consistent with the model-derived phase estimates.

### 6.2. Averaging over structure-factor trajectories following convergence to improve the solution estimate

The results obtained (Fig. 8 ▸, Supplementary Movie S1) suggest that averaging over the stationary region of the algorithm trajectory, following convergence to the solution, could be used to improve the estimate of the electron density. Such averaging could be performed in either real or Fourier space, and we have investigated the latter. This kind of averaging operation has a precedent in the field of coherent X-ray imaging (Shapiro *et al.*, 2005 ▸; Thibault *et al.*, 2006 ▸).

We hypothesized that the trajectory of the individual Fourier coefficients generated by the IPA, following global convergence to the solution (Fig. 8 ▸), might be reflective of the probability distributions for each Fourier coefficient arising from imposition of the constraints. Hence, it could be useful to estimate from each trajectory the components of the first trigonometric moment of the phase-angle distribution. When expressed in polar form these are the mean direction (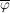) (equation 20) and mean length (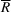) (equation 21). In the absence of amplitude error, the best Fourier synthesis, in a least-squares sense, could then be calculated using the mean direction (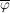) as the phase, while weighting the Fourier amplitudes by the mean length (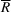) (Read, 1997 ▸; Barnett & Kingston, 2024 ▸). However, even if the structure-factor distributions obtained following convergence cannot be interpreted in this way, it is apparent, by inspection, that simply using the mean direction as the phase together with the unweighted Fourier amplitudes should yield an improved estimate of the electron density.

To explore this hypothesis, we performed phase-retrieval runs for a number of test cases using the RRR algorithm. The experiments were performed with β ranging from 0.3 to 1.1, which corresponds to the values shown earlier to be most effective for phase retrieval (Section 5.2). At each value of β tested, 30 runs. each of 150 iterations, were performed. Each run was initiated with model-derived phases corrupted with random error (circular variance of the error functions *V* = 0.75, corresponding to a mean absolute phase difference of ∼70° with the model phases). The molecular envelope was estimated from the solution estimate at each iteration.

**Figure 9:**
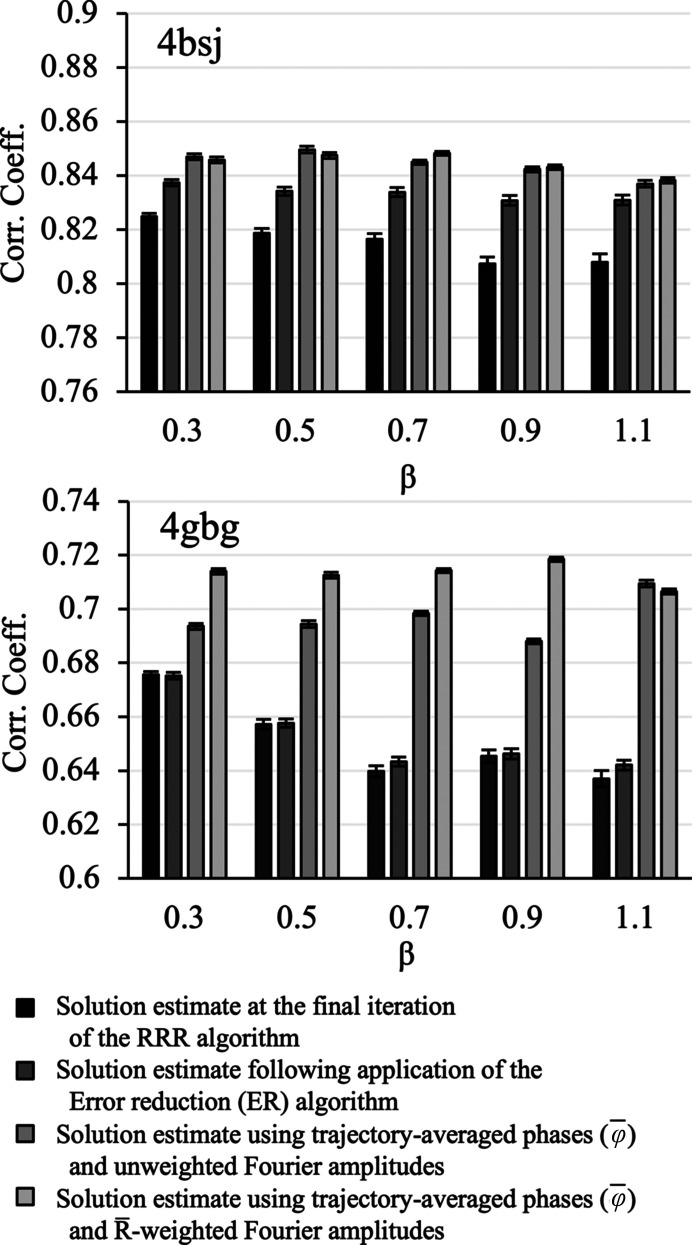
Accuracy of density estimates obtained by averaging over the RRR algorithm trajectory following convergence to the solution versus application of the ER algorithm to improve the final solution estimate for test cases 4bsj and 4gbg. Results are reported as a function of the RRR algorithm parameter β. Computational procedures are indicated in the key. The mean real-space map correlation with the known solution (over 30 replicates) is indicated by the bar height. The associated error bars show the standard error of the mean.

From the trajectories of the 30 replicates, all of which converged to the solution, we calculated the sample mean direction (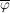) and mean length (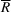) of the phase-angle distribution for each term *hkl* using equations (18)–(21) over a window of 30 iterations extending backwards from the end of the run. This nonparametric procedure is applicable to both the centric and acentric data. Electron-density maps were subsequently computed using the mean direction (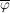) as the phase, in combination with either unweighted Fourier amplitudes or Fourier amplitudes weighted by the mean length of the phase-angle distribution (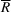). We compared the results with an alternate procedure in which the solution estimate at the final iteration of the RRR algorithm was subjected to an additional 30 iterations of the ER algorithm to damp fluctuations in the estimate. We have previously used this procedure to improve the phase and density estimates at the end of a phase-retrieval run (Kingston & Millane, 2022 ▸). We then assessed the mean agreement of each of the resulting density functions with the map derived from the atomic model. The results are shown in Fig. 9 ▸ for two test cases (PDB entries 4bsj and 4gbg) which are representative of the results obtained.

For the RRR algorithm, the breadth of the structure-factor distributions observed in the complex plane increases with β in a quite regular fashion (Supplementary Fig. S3), as β controls the step size of the algorithm (equation 4*a*[Disp-formula fd5]). In other words, as β increases the RRR algorithm effectively samples from increasingly broad structure-factor distributions following convergence to the solution (or equivalently, the fluctuations in the electron-density function become steadily larger). Therefore, the single-point solution estimate obtained at the final iterate of the RRR algorithm becomes steadily worse with increasing β in all cases (Fig. 9 ▸). However, averaging over the structure-factor trajectories generated by the algorithm in the stationary region (*i.e.* using the mean direction, computed from the stationary part of the algorithm trajectory, as the phase estimate) is effective in improving the solution estimate at each value of β. The averaging appears to be uniformly effective at lower values of β (0.3–0.7). In some cases, it appears significantly better to weight the Fourier amplitudes by the mean length of the phase-angle distribution (Fig. 9 ▸, test case 4gbg), while in other cases the results of this procedure are comparable or slightly worse than those obtained using unweighted Fourier amplitudes (Fig. 9 ▸, test case 4bsj). Both averaging procedures routinely outperform the alternative, which is to apply the ER algorithm to improve the final density estimate. While application of the ER algorithm might be expected to drive the phases to the long-term averages apparent in the trajectory of the RRR algorithm, its poor global convergence properties mean that this is only partially achieved. As β increases and the density estimate at the final iterate becomes worse, this becomes increasingly problematic, and the result returned by applying the ER algorithm degrades. For the specific case of the RRR algorithm, where the variance in the solution estimate responds so regularly to β (Supplementary Fig. S3), it would also be possible to improve the solution estimate by systematically reducing β towards the end of the run. However, this would appear to have no advantage over averaging across the solution trajectory in the stationary region.

Similar outcomes to those obtained above might also be achieved by averaging the final outputs of multiple independent phase-determination runs, as we have performed previously when using the DM algorithm (Kingston & Millane, 2022 ▸). However, the present procedure, which exploits the information contained in the trajectory of a single run once it has become stationary, is far more computationally efficient.

### 6.3. Phase uncertainty as a function of resolution

The uncertainty in the phases estimated by IPAs is very dependent on the Fourier amplitude (Fig. 8 ▸, Supplementary Movie S1) and hence on the resolution. As the resolution increases, the precision of the phase estimates decreases. To capture the resolution-dependence of the phase-retrieval process, the phase-retrieval transfer function (PRTF) was introduced in the field of coherent X-ray imaging (Chapman *et al.*, 2006 ▸). The PRTF is defined as the amplitude of the averaged complex Fourier coefficients obtained from multiple solution estimates, normalized by the experimental Fourier amplitudes. In a crystallographic setting, this statistic can be straightforwardly calculated from the trajectory of an IPA when it is stationary (equation 23). We note that for the RRR algorithm, averaging the complex structure factors over some part of the trajectory is either exactly (solution estimate given by equation 4*c*[Disp-formula fd6]) or nearly (solution estimate given by equation 4*b*[Disp-formula fd6]) equivalent to weighting the measured amplitudes by the mean length of the phase-angle distribution, as we have performed in Section 6.2. In other words, in computing the PRTF from the RRR algorithm trajectory, each term in the numerator is essentially the Fourier amplitude weighted according to the confidence with which its phase is known. Consequently, if there is no uncertainly in the determined phases (they have a single-point distribution) the PRTF will evaluate close to 1, while if the determined phases are random (they have a uniform circular distribution for the acentric data) the PRTF will evaluate to 0.

**Figure 10:**
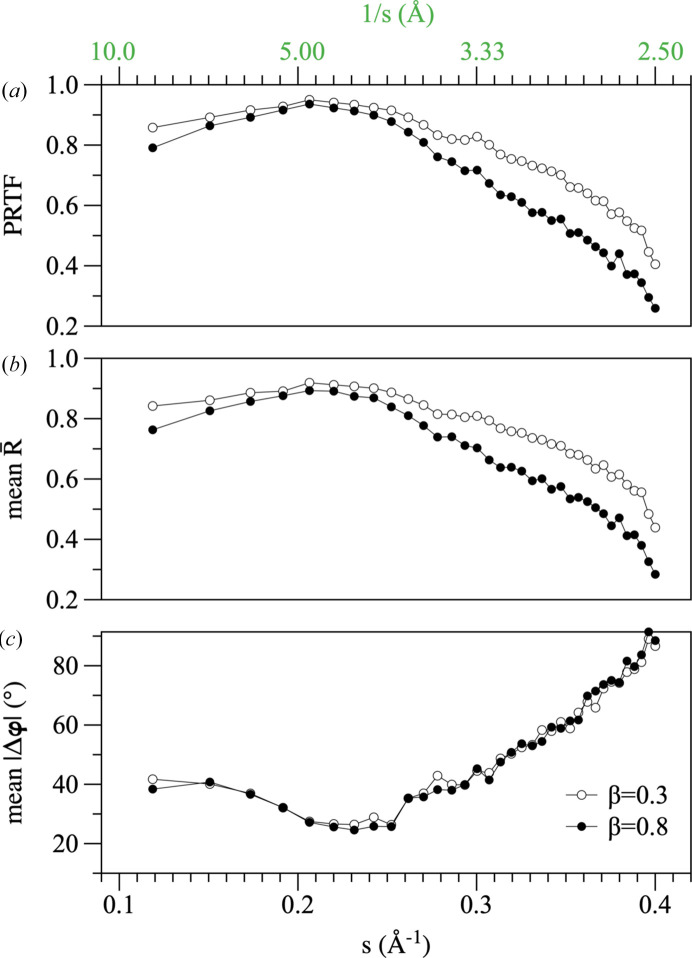
Resolution-dependence of the phase uncertainty inferred from trajectory averaging for test case 4bsj. (*a*) The phase-retrieval transfer function (PRTF) as a function of resolution (1/*s*). (*b*) The mean mean length (mean 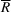) of the phase-angle distributions as a function of resolution (1/*s*). (*c*) The mean absolute difference (mean |Δφ|) between the trajectory-averaged and model phases as a function of resolution (1/*s*). The statistics were evaluated over 30 iterations of the RRR algorithm (β = 0.8 or β = 0.3 as indicated) once the algorithm had become stationary.

The PRTF calculated for test case 4bsj, and averaged within concentric resolution shells, is shown in Fig. 10 ▸(*a*) together with the mean length (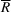) of the phase-angle distributions (Fig. 10 ▸*b*) and the absolute difference |Δφ| from the known phases (Fig. 10 ▸*c*), averaged in the same fashion. As expected, the PRTF and the mean length of the phase-angle distributions provide effectively the same information about the decrease in phase reliability with resolution, although in involving the Fourier amplitudes, the PRTF is the more physically informative statistic. While it has been suggested that the point at which the PRTF drops below some empirical threshold (typically 0.5) might be used as an objective estimate of image resolution, the circular variance of the phase-angle distributions (and hence the absolute value of the PRTF) is dependent on the RRR algorithm parameter β (Figs. 10 ▸*a* and 10 ▸*b*). In contrast, the mean direction of the phase-angle distributions is essentially unchanged with β, and hence the phase sets are equally accurate in each case (Fig. 10 ▸*c*). Hence, the PRTF cannot yield absolute estimates of image resolution until the connections between the phase-angle distributions generated by the algorithm and the constraints on the solution are better understood. This is in concordance with the results of the previous section (refer to Supplementary Fig. S3). However, the PRTF is certainly informative of relative phase uncertainty as a function of scattering angle, and this has physical relevance, as comparison of Figs. 10 ▸(*a*) and 10 ▸(*c*) makes clear.

## 7. Conclusion

In this paper, we have explored the behavior of a number of iterative projection algorithms for crystallographic phase retrieval. We have emphasized the practical application of the algorithms, rather than the theoretical consideration of their properties, which have been discussed extensively elsewhere (Marchesini, 2007 ▸; Millane & Lo, 2013 ▸; Millane, 2023 ▸). The real-space constraints employed were solvent flatness and histogram equivalence. Although we do not explore the issue in this paper, it is easy to demonstrate, both theoretically and practically, that the solvent-flatness constraint is by far the more powerful of the two constraints employed.

Previously, we have used the DM algorithm (equation 3*a*[Disp-formula fd3]) to develop a direct phase-determination procedure for high-solvent-content crystals (Kingston & Millane, 2022 ▸), illustrating the potential of IPAs for *ab initio* phase retrieval. Several alternatives to the DM algorithm have been developed, which have seen little or no use in the crystallographic setting. Here, we have performed some comparative experiments using two such alternatives: the RRR algorithm (equation 4*a*[Disp-formula fd5]; Elser *et al.*, 2018 ▸) and a reversed variant (equation 5*a*[Disp-formula fd7]), and the RAAR algorithm (equation 6; Luke, 2005 ▸). The results (Figs. 3 ▸, 4 ▸, 5 ▸ and 6 ▸, Supplementary Table S1) demonstrate that all of these algorithms appear to be effective for crystallographic phase retrieval, given appropriate values of their adjustable parameter β. There may be computational advantages associated with the choice of algorithm. For example, the performance of the RRR algorithm appears to be rather insensitive to the exact value of β (Fig. 4 ▸) and it is less costly to evaluate than the DM algorithm.

A property of all of these constraint-satisfaction algorithms (DM, RRR and RAAR) is that even when they have arrived at the solution, and the algorithm trajectory is stationary, the density function, and hence the associated Fourier coefficients, continue to significantly fluctuate around the mean value (Figs. 7 ▸ and 8 ▸, Supplementary Movie S1) because it is not possible to exactly and simultaneously satisfy all of the constraints. We show that averaging across the stationary part of the trajectory, which has negligible computational cost, can be used to improve the solution estimate (Fig. 9 ▸). We have performed this averaging operation in Fourier space, estimating the first trigonometric moment of the phase-angle distribution for each Fourier coefficient from the algorithm trajectory following convergence and then incorporating this information into the Fourier synthesis in the usual way. The resulting map is significantly better than that obtained by simply taking the output of the algorithm at the final iteration, and is also generally better than that obtained by applying the ER algorithm to improve the final iterate, as we and others have done in the past. Trajectory averaging has therefore been incorporated as the default procedure in our program for direct crystallographic phase retrieval (*IPA*).

There are theoretical issues which remain to be explored. In particular, the relationship between the phase distributions derived from the algorithm trajectory and the real-space constraints being applied needs to be systematically investigated. Our current treatment of these frequency distributions as ‘probabilities’ is practically effective (Fig. 9 ▸) but purely empirical. If this limitation can be addressed, then trajectory averaging might additionally be used to generate reliable resolution estimates via analysis of the PRTF (Fig. 10 ▸). However, even at the current state of development, our results suggest that switching from a deterministic to a probabilistic view of phase determination when using iterative projection algorithms is likely to prove productive, just as it has been for the procedures involved in experimental phase determination (Read, 2003 ▸; Bricogne *et al.*, 2003 ▸; McCoy & Read, 2010 ▸).

One place in which a probabilistic framework might be applied is understanding how the real-space constraints on the density function propagate into Fourier space. The constraints being applied to the density function could be expressed in the Fourier domain as a system of nonlinear equations (Main, 1990 ▸). Such a system of equations can in principle be analyzed to understand the phase constraints existing on the system. This kind of approach was previously adopted to analyze the phase restrictions resulting from the presence of noncrystallographic symmetry (Main & Rossmann, 1966 ▸; Crowther, 1967 ▸; Main, 1967 ▸; Crowther, 1969 ▸). The work presented here suggests that iterative projection algorithms may ultimately provide a more computationally convenient way to address this same problem, through investigation of the impact of the real-space constraints on the phase-angle distributions generated by the algorithms.

## Supplementary Material

Supplementary Figures and Table and caption to Supplementary Movie S1. DOI: 10.1107/S2059798324009902/nz5017sup1.pdf

Supplementary Movie S1. DOI: 10.1107/S2059798324009902/nz5017sup2.mov

## Figures and Tables

**Figure 5:**
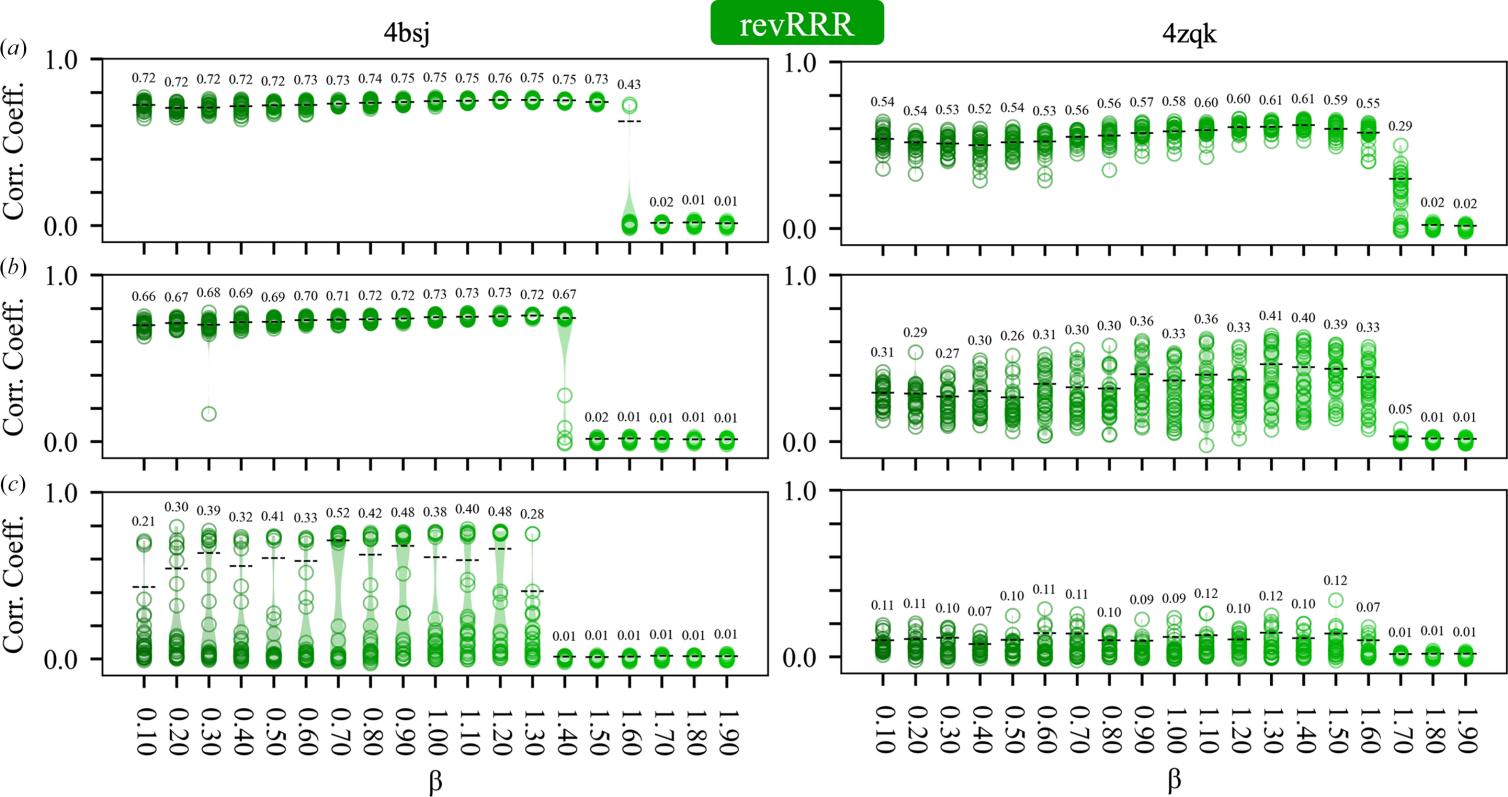
Performance of the revRRR algorithm as a function of its adjustable parameter β for test cases 4bsj and 4zqk. Details are as for Fig. 3 ▸.

**Figure 6:**
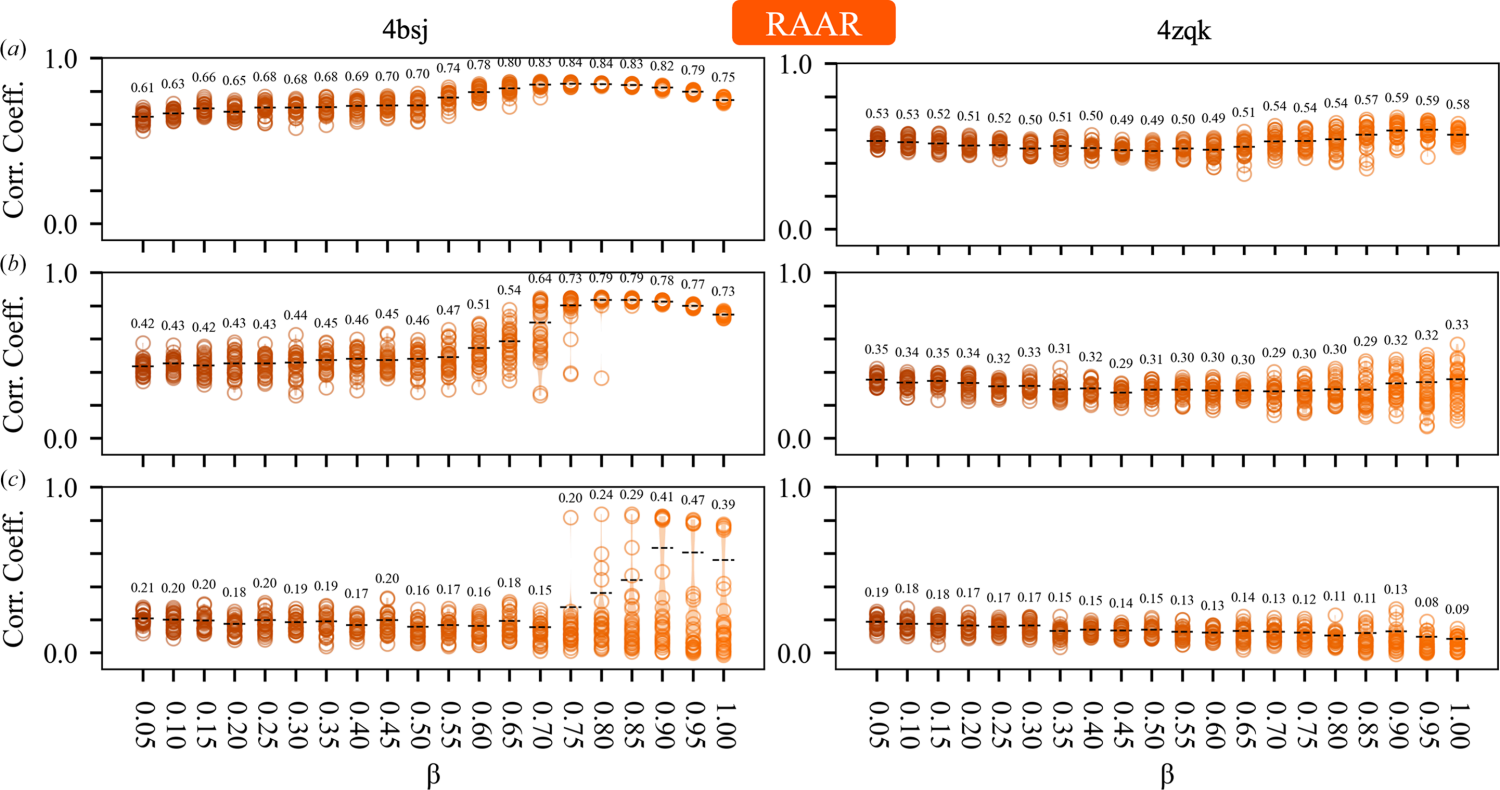
Performance of the RAAR algorithm as a function of its adjustable parameter β for test cases 4bsj and 4zqk. Details are as for Fig. 3 ▸.

**Table 1:** Crystallographic data used to generate the figures

Protein Data Bank (PDB) identifier	Protein	Resolution (Å)	Solvent fraction	Reference
4bsj	Human vascular endothelial growth factor 3 (VEGF-3) extracellular domains	2.5	0.74	Leppänen *et al.* (2013 ▸)
4zqk	Complex of human programmed death-1 (PD-1) and its ligand PD-L1	2.45	0.61	Zak *et al.* (2015 ▸)
4nli	Ovine β-lactoglobulin	1.9	0.76	Loch *et al.* (2014 ▸)
4gbg	*Thermomyces lanuginosa* lipase	2.9	0.68	S. Yamini, J. Mukherjee, M. N. Gupta, M. Sinha, P. Kaur, S. Sharma & T. P. Singh (unpublished work)
