# Supplementary material for: Analysis of crystallographic phase retrieval using iterative projection algorithms
Source: Acta Crystallogr D Struct Biol. 2024 Oct 23;80(Pt 11):800–18. doi: 10.1107/S2059798324009902 (PMC11544429; doi:10.1107/S2059798324009902)
Supplement: Supplementary file 1 [file d-80-00800-sup1.pdf]

STRUCTURAL BIOLOGY

Volume 80 (2024)

Supporting information for article:

Analysis of crystallographic phase retrieval using iterative projection algorithms

Michael J. Barnett, Rick P. Millane and Richard L. Kingston

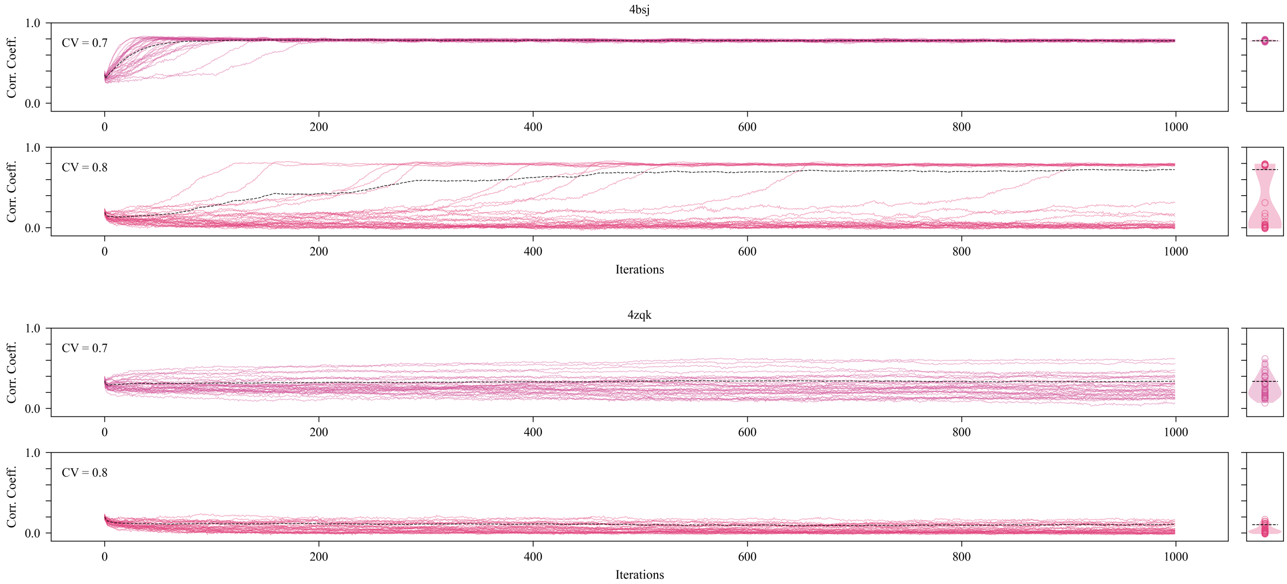

Figure S1 A replicate of the experiment shown in Fig. 2 (c) and (d) of the main paper, in which 1000 iterations are completed.

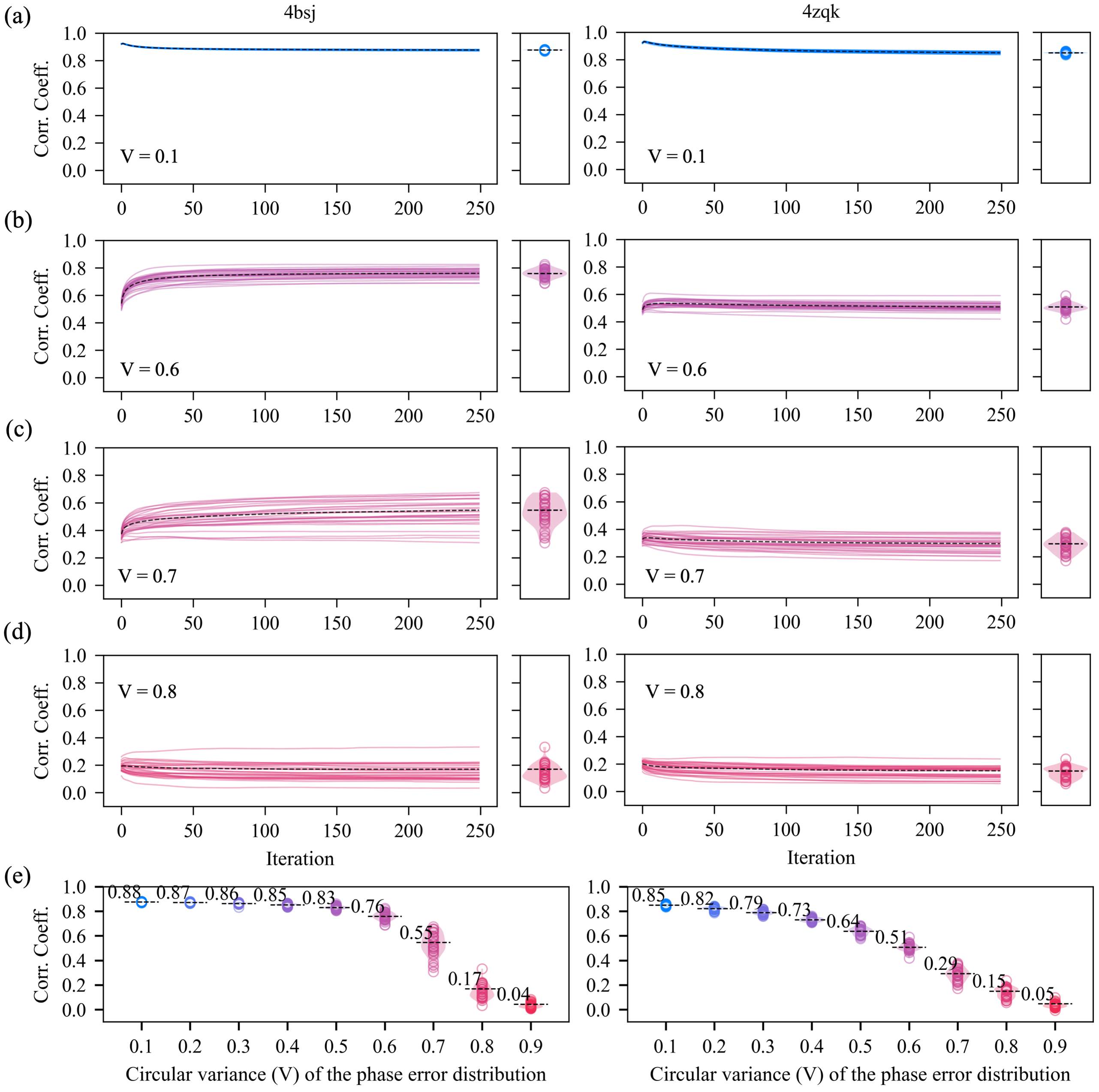

Figure S2. A replicate of the experiment shown in Fig. 2 of the main paper, but using the error reduction (ER) algorithm instead of the difference map (DM) algorithm (β=0.75)

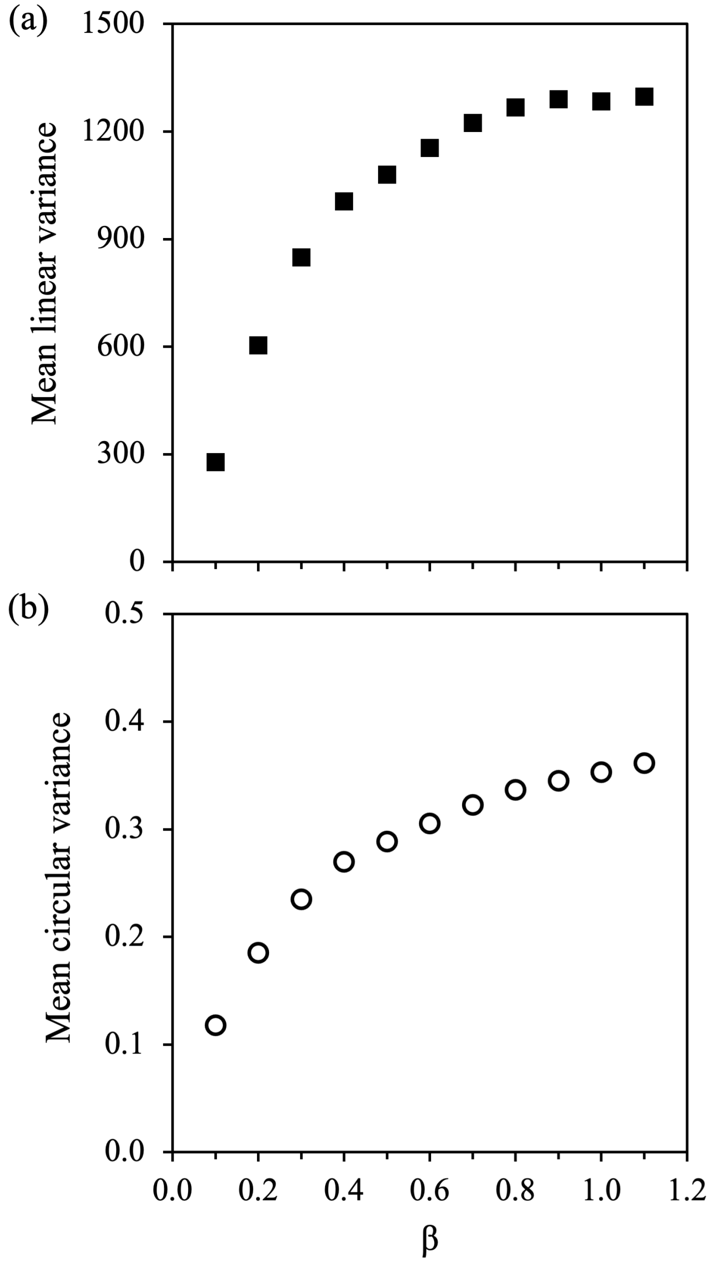

Figure S3 Mean width of the structure factor distributions generated by the RRR algorithm when converged to the solution (test case 4nli), as a function of algorithm parameter β. Shown are (a) the mean linear variance of the amplitude distributions, and (b) the mean circular variance of the phase distributions associated with the solution estimate (4b), and computed from all data. The mean variances of the Fourier coefficients were evaluated from 8 independent runs over a window of 30 iterations, once the algorithm was stationary. Each run was initialised with model-derived phases corrupted with a small amount of random error (circular variance of the error functions V = 0.1, corresponding to a mean absolute phase difference of ~20° with the model phases) to allow rapid attainment of stationarity.

Table S1. Comparative performance of IPAs for direct phase retrieval. Shown are the fraction of runs for which the solution was successfully located (real space density correlation with known solution > 0.75) for five different test cases.

Test Case	Algorithm				
**PDB ID** / Solvent Fraction / Resolution(Å) (Correlation coefficient between initiating and known molecular envelope)	**DM** β=0.75)	**DM** β=-0.9)	**RRR** (β=0.7)	**RevRRR** (β=0.7)	**RAAR** (β=0.9)
**3mf0** / 0.71 / 3.10 (0.22)	0/20	0/20	0/20	4/20	0/20
**4bsj** / 0.74 / 2.50 (0.31)	3/20	0/20	3/20	2/20	7/20
**1dov** / 0.77 / 3.00 (0.55)	13/20	5/20	10/20	10/20	1/20
**2ja1** / 0.77 / 2.80 (0.79)	13/20	16/20	9/20	9/20	10/20
**4fzn** / 0.80 / 2.86 (0.87)	11/20	6/20	2/20	6/20	13/20

Each algorithm was executed twenty times for each test case, beginning with the same twenty randomly-generated phase sets, and was allowed to progress for 8040 Iterations. For each test case a low resolution molecular envelope was generated using the RRR algorithm and used to initiate all the phase retrieval runs (the Matthews correlation coefficient between initiating and known envelope is indicated).

Movie S1. Location of the solution to the phase retrieval problem using the RRR algorithm (β=0.80, test case 4nli).

(a) The trajectory of the electron density function, with the known atomic model displayed in ball and stick representation.

(b) The circular variance of the phase angle distributions together with deviations from model-calculated phases, plotted as a function of Fourier amplitude for all acentric terms. The sample mean length and mean direction of the phase angle distribution were calculated over a window of 30 iterations, extending backward from the current iteration. The circular variance (1 - mean length) for each term is plotted as a single small circle, with the color of the circle indicating the absolute difference between the mean direction and the model-calculated phase, as shown in the key. The slightly larger circles correspond to observations with indices h=8, k=6, l=24 (left) and h=8, k=2, l=4 (right) whose full trajectories are shown in panel(d). The mean circular variance calculated from all acentric terms is shown as a horizontal dashed line.

(c) The evolution of the real space correlation coefficient between the solution estimate and the density calculated from the known atomic model as well as the mean circular variance of the phase angle distributions for the acentric Fourier coefficients.

(d) The trajectory of two individual Fourier coefficients, plotted in the complex plane, as in Figs 7 and 8, together with a bivariate probability density function (17) fit over a window of 30 iterations, extending backward from the current iteration. The displayed iso-contours of the fitted PDF pass through $\mu_G \pm 1\sigma$, $\mu_G \pm 2\sigma$ and $\mu_G \pm 3\sigma$, along the central symmetry axis of the distribution.
